# Utilizing machine learning for survival analysis to identify risk factors for COVID-19 intensive care unit admission: A retrospective cohort study from the United Arab Emirates

**DOI:** 10.1371/journal.pone.0291373

**Published:** 2024-01-11

**Authors:** Aamna AlShehhi, Taleb M. Almansoori, Ahmed R. Alsuwaidi, Hiba Alblooshi

**Affiliations:** 1 Biomedical Engineering Department,College of Engineering, Khalifa University, Abu Dhabi, United Arab Emirates; 2 Healthcare Engineering Innovation Center (HEIC), Khalifa University, Abu Dhabi, United Arab Emirates; 3 Department of Radiology, College of Medicine and Health Sciences, United Arab Emirates University, Al Ain, United Arab Emirates; 4 Department of Pediatrics, College of Medicine and Health Sciences, United Arab Emirates University, Al Ain, United Arab Emirates; 5 Department of Genetics and Genomics, College of Medicine and Health Sciences, United Arab Emirates University, Al Ain, United Arab Emirates; Universidad de La Sabana, COLOMBIA

## Abstract

**Background:**

The current situation of the unprecedented COVID-19 pandemic leverages Artificial Intelligence (AI) as an innovative tool for addressing the evolving clinical challenges. An example is utilizing Machine Learning (ML) models—a subfield of AI that take advantage of observational data/Electronic Health Records (EHRs) to support clinical decision-making for COVID-19 cases. This study aimed to evaluate the clinical characteristics and risk factors for COVID-19 patients in the United Arab Emirates utilizing EHRs and ML for survival analysis models.

**Methods:**

We tested various ML models for survival analysis in this work we trained those models using a different subset of features extracted by several feature selection methods. Finally, the best model was evaluated and interpreted using goodness-of-fit based on calibration curves,Partial Dependence Plots and concordance index.

**Results:**

The risk of severe disease increases with elevated levels of C-reactive protein, ferritin, lactate dehydrogenase, Modified Early Warning Score, respiratory rate and troponin. The risk also increases with hypokalemia, oxygen desaturation and lower estimated glomerular filtration rate and hypocalcemia and lymphopenia.

**Conclusion:**

Analyzing clinical data using AI models can provide vital information for clinician to measure the risk of morbidity and mortality of COVID-19 patients. Further validation is crucial to implement the model in real clinical settings.

## Introduction

The COVID-19 pandemic started first in Wuhan (China) in December 2019 and has expanded to every inhabited continent [1, 2]. In March 2020, the World Health Organization (WHO) categorized it as a global pandemic with remarkably high incidence and mortality rates [1–3] and to date, 683,955,862 total number of infected individual with 6,831,756 tolls of death. Until now, supportive care is the main treatment available. Different COVID-19 vaccines were developed and are currently used to reduce the susceptibility to infection [1].

The pandemic has drastically challenged the health care systems globally. The increment in the number of affected individuals exerted substantial pressure on health sectors particularly with the limitation in the intensive care units (ICU) [2]. The main challenge for health professionals was to identify the cases that are more likely to progress from mild to severe or sudden death during the early stages of the pandemic. Understanding the risk factors for severe disease can help the clinician to provide a timely and efficient intervention including proper utilization of ICU facilities.

Since the beginning of the pandemic, tremendous quantitative research using Electronic Health Records (EHRs) were undertaken for different objectives such as patients discharge time prediction [2], mortality risk prediction [4] and early-detection models for COVID-19 [5]. Several studies reported the clinical characteristics and relevant risk factors for severe disease. In a Malaysian cohort [6], chronic kidney disease, chronic pulmonary disease, fever, cough, diarrhea, breathlessness, tachypnoea, abnormal chest radiographs and high serum CRP (≥5 mg/dL) at the time of hospital admission were found as risk factors for severe disease using univariate and multivariate logistic regressions for 5,889 confirmed COVID-19 patients. Another study of 17,278,392 patients from England [3], reported gender, age, diabetes and asthma to be associated with severe COVID-19 cases using multivariable Cox proportional hazards model. A second retrospective study from England [7] (n = 3,138,410) focusing on diabetic population, showed that severity of COVID-19 disease was associated with history of cardiovascular disease, gender, age, renal impairment, non-white ethnicity, socioeconomic deprivation, poor glycemic control and high body mass index (BMI) using Cox proportional hazards model. A Scotland representative cohort [8] also for diabatic patients consists of 5,463,300 patients reported using logistic regression that the risks factors that associate with fatal or critical care unit-treated COVID-19 are: gender, smoking, live in residential care, retinopathy, reduced renal function, or worse glycemic control, diabetic ketoacidosis or hypoglycemia. A retrospective study of Kazakhstan diabetic population [1] (n = 1961) showed that the severity of COVID-19 was higher in diabetic patients, in which they have higher rates of coexisting cardiovascular pathology and kidney disease. Also, the clinical symptoms such as impaired breathing and nausea/vomiting and weakness/lethargy are worst in this group in comparison to the non-diabetic matched group. The previous works reported several risk factors that are associated with COVID-19 severity. A common risk factors for severe cases are age and gender. Those risk factors are various across countries.

This study aims to explore and report the association between patient’s hospital admission medical information and the patient’s deterioration to access the ICU. To the best of our knowledge, this is the first quantitative study employing machine learning for survival analysis to report risk factors to access the ICU in a United Arab Emirates (UAE) cohort. The cross-validation predictive model of accessing critical care unit for COVID-19 patients is also explored.

## Materials and methods

### Ethical statement

This study was approved by the Institutional Review Board of the Department of Health, Abu Dhabi. Approval number: IRB DOH/CVDC/2020/799. The review board waived the requirement for individual informed consent as this study was part of outbreak investigation in the UAE. All investigators had access to only anonymized patient information. This study was performed in accordance with the relevant laws and regulations that govern research in the emirates of Abu Dhabi, UAE.

### Data source

In this retrospective study, an anonymized COVID-19 patients’ medical records extracted from the Abu Dhabi Health Services Company (SEHA) healthcare system. SEHA dataset is a high-dimensional UAE population health data source which include rich patients’ medical information such as sociodemographic, comorbidity, upon admission: symptoms, laboratory results, vital signs, and COVID-19 medications.

### Study design

**Fig 1:**
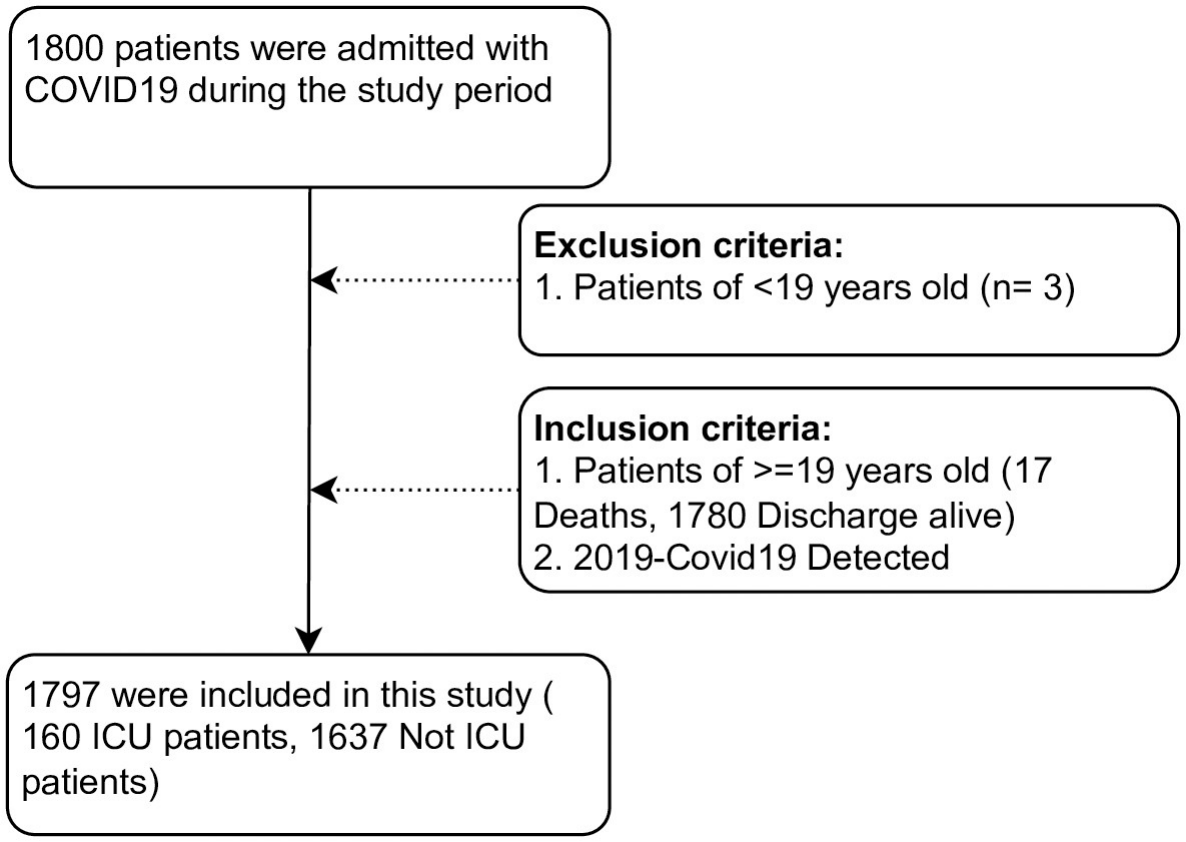
Flow diagram of the cohort. Inclusion and exclusion criteria for determining patients consider in this study. SEHA extracted 1,800 COVID-19 patients from March 1, 2020 to April 20, 2020. After applying the inclusion and exclusion criteria, our study included 1787 patients.

A COVID-19 cohort was identified and extracted by SEHA. The clinical diagnosis of COVID-19 was confirmed using Reverse-transcriptase-polymerase chain reaction (RT-PCR) from nasal swabs. The data contained 1,800 registered patients (ages ≥ 18 years) admitted to any of SEHA healthcare facilities between March 1, 2020, and April 20, 2020. We excluded patients who were under 19 years of age following Petrilli et al. [9]; Fig 1 shows the flow diagram of the cohort inclusion and exclusion criteria. Patients were divided into two groups with respect to severity by accessing the ICU unit. The follow-up period started from the date of hospital admission up to the date of ICU access or date of discharge from the hospital. Some patients had up to 60 days of follow-up. We extracted all patients’ baseline (upon admission) covariates provided in the dataset.

### Modeling

EHRs data contain rich information of patients’ medical information. Those patient-centered records impose challenges because of its heterogeneous, high-dimensional nature as well as the presence of missing and censored records [10]. These various challenges require advanced techniques to detect the patterns and report unseen association between the variables of interest and the outcome. In the following section, we will outline the study pipeline to address the challenges imposed by such complex data and how we built a robust model to report the association between patient baseline information and developing a critical situation leading to ICU admission.

#### Data pre-processing

Missing value is a common problem in a medical data such as electronic health records, which needs to be handled in a proper way to avoid biased results [11]. We encountered this problem in our dataset; variables with missing variables are reported in the Supplementary S1 to S6 Figs. To deal with this problem, we first excluded the variables with more than 70% missing information and imputed the remaining variables. Understanding and describing the missingness mechanism is an important step in determining the best way to handle them. The pattern of missingness was performed using the pairs plots to assess the relationships between missing values and observed values in all variables [12] (S7 to S17 Figs). From the pattern analysis, we conclude that the missingness is Missing at random (MAR). Therefore, Multiple imputation using random forest was employed in this study. Multiple imputation using random forest is the most popular and robust imputation method widely used for this task [11] (S18 Fig).

#### Baseline characteristics statistical analysis

Differences in the baseline patient’s information grouping by accessing the ICU variable were tested using the *t*-test for parametric continuous variables (with equal variance assumption), while the Mann-Whitney U test was used for nonparametric continuous variables. The *χ*^2^ test was used to test categorical variables hypotheses (with continuity correction), while Fisher’s exact test was used for smaller sample sizes (small cell counts). All the statistical analyses were tested at the 95% significance level.

#### Statistical and machine learning models

1. Feature Selection: To address the high-dimensional problem in our dataset, we utilized several feature selections approaches to find the prominent subsets of feature to train our final models. Feature selection assisted to speed up the training time, enhance the model interpretability, and improve model performance. The following are the methods/approaches used in this study:
  - Random forest variable importance (RF Var Imp): This method relies on random permutation of the feature to calculate its importance. In which model performance measure after and before the imputation. If the model prediction error increase significantly after the imputation, it will assign an importance score to the feature which reflects how important it is to the final prediction [10].
  - Random forest minimal depth (RF Min Depth): The method measures the shortest distance between the tree main root to the largest subtree. The feature of interest is its root. The shortest the distance the more influent and significant the feature is [10].
  - Univariate Score using Survival tree: The model fits survival tree for each single feature in turn and the importance of the feature determine by prediction for the survival tree model.
  - Cox model permutation importance (CPH Perm Imp): Similar to, RF Var Imp but random forests model substitute with Cox model.
  During this process and specifically for machine learning based features selection, we selected the optimal subset of features by keeping specific percentage of the highest importance features reported by the model. The optimal percentage threshold value selected using 5-fold cross-validation.
2. Survival Analysis Models: Survival analysis is a subfield from statistical methods which focus on modeling the time to event or the expected time until the event of interest occurs. The event of interest might not be observed or missed during the study period for some patients; those data points named censored data [2, 10]. Survival analysis approach was performed in this study rather than a classification approach. In the classification analysis we have to train the model at each time step. Consequently, classifier can only determine whether or not the patients will experience the event of interest without knowing when exactly the event will occur. Whares, in survival analysis consider the time till the event occur [13]. One of most popular survival analysis models in clinical study is Cox Proportional Hazard Model which lacks the scalability to high-dimensional data [10]. Recently, many machine learning methods were adopted for survival analysis to incorporate the capability of such advanced models to handle complex relation between variables and its capability to scale for high-dimensional data [10]. Some machine learning for survival analysis are Survival Tree, Gradient Boosting Machine, Random Forests and Regularized Generalized Linear Model. Following is a detailed description of the statistical and machine learning survival models considered in this study:
  - Multivariate Cox Proportional Hazard (CPH) Model is a standard and most popular survival analysis model in medical domains [14]. It is fast, computation unexpensive and easy to use and interpret. However, CPH has several drawbacks such as its inability to deal with high dimensional dataset, and to model the nonlinear interaction and correlation between the features; finally, CPH model makes several assumptions that need to be satisfied to produce valid results such as proportional hazards assumption, test for influential observations, and nonlinearity [14].
  - Survival Tree is a tree-base method like the tradition decision tree machine learning method. The model recursively partition tree nodes based on splitting rules that incorporate censoring information such as log-rank which is the most popular splitting rules for survival model [13].
  - Random Forests (RFs) are the extended version of traditional random forests which incorporate the censored information during mode training. RFs is an ensemble tree-based model; each individual decision tree divided its nodes based on splitting rule that incorporate censoring information such as log-rank splitting and gradient-based brier score. The final outcome is calculated by averaging the predicators of each tree [14].
  - Regularized Generalized Linear Model (GLM): fits a regularized Cox model using a penalized negative log of the partial likelihood with an elastic net penalty. This model adds a regularization term to control the overfitting problem and reduce model complexity. Three GLMs can be fitted depending on the alpha(*α*) values: elastic net, ridge, and lasso. Elastic net model combines and bridges the gap between the other two penalties models: ridge (*α* = 0) and lasso (*α* = 1) [15].
  - Gradient Boosting Machines (GBMs) are a stage-wise models which convert a weak learner (tree-based) into a stronger model by incorporating optimization function such as gradient descent to minimize the objective (loss) function [14].
3. Hyperparameters Optimization: Machine learning models hyperparameters tuning with random search optimization algorithm applied to choose the optimal set of models’ parameters that yield the best performance. We used 5-fold cross-validation to assess the selected parameters quality. Table 4 presents the parameter search space of each model and the selected parameter for the final selected models.
4. Model Evaluation and Explanation
  - Discrimination (Concordance Index): model performances were compared and measured using Concordance index (C-index). C-index is a standard evaluation measure for survival analysis, it measures the proportion of the concordant pairs between all possible evaluation pairs [2, 14].
  - Partial Dependence Plots (PDPs): model interoperation and transparency is an important factor to adopt machine learning for clinical practice [14]. In this study, we applied PDPs as a post hoc technique to explain model decisions. The plots showed the marginal effect of feature of interest as a risk factor on the outcome of interest [15].
  - Calibration curve: a plot representation of the model-predicted probabilities versus observed event rates within a given duration. Survival probability is ranked first, followed by partitioning the data set into groups. The subjects in the upper group are those who are least likely to experience the event of interest, while those in the lower group are most likely to experience it [16].
  - Models’ comparison: We compare the performance of different algorithms based on several runs with shuffling using the Kruskal-Wallis test, a non-parametric method for comparing distributions of model outcomes (C-Index). Then we perform a pairwise comparison among the different models using the Nemenyi posthoc test to detect the models that differ from each other.

## Results

### Baseline characteristics statistical analysis

Baseline sociodemographic, comorbidity, upon admission: symptoms, laboratory results, vital information, and the descriptive statistics are presented in Tables 1–3 for the 60 features included in this study. In general, the non-ICU group was younger and had a larger number of patients than the ICU group. Also, the majority of the population is male in both groups, with no significant difference between them. Several features were significantly different between the two groups namely, age, diet, obesity, diabetes, CKD, ESRD, cough, c reactive protein, calcium level, chloride level, CO2 level, creatinine, ferritin level etc. We applied a correlation matrix to identify and remove the highly correlated independent variables (greater than 0.7 and less than -0.7) and we end up with 55 features for training the different models; the heat map for the correlation matrix is found in (S19 Fig).

**Table 1:** Baseline characteristics statistical analysis: Baseline characteristics of patients stratified by severity measure by accessing the ICU, mean (SD) or N (%).

	ICU	NON-ICU
N	160	1637
Age (yrs.)		*
[19,45)	76 (47.5)	1209 (73.9)
[45,55)	41 (25.6)	308 (18.8)
[55,65)	31 (19.4)	103 (6.3)
[65,75)	10 (6.2)	15 (0.9)
[75,85)	2 (1.2)	2 (0.1)
Male	139 (86.9)	1383 (84.5)
Nationality		
UAE National	6 (3.8)	111 (6.8)
Gulf Cooperation Council countries (GCC)**	0 (0.0)	18 (1.1)
Others	154 (96.2)	1508 (92.1)
Pregnancy (Yes (%))	2 (1.2)	6 (0.4)
Diet		*
Anti-Diarrhea Diet	0 (0.0)	7 (0.4)
Cardiac Diet	5 (3.1)	10 (0.6)
Diabetic Diet	29 (18.1)	136 (8.3)
Enteral Feeds	17 (9.4)	6 (0.3)
Full Liquid Diet	2 (1.2)	1 (0.1)
G6PD Diet	0 (0.0)	7 (0.4)
Hepatic Diet	0 (0.0)	1 (0.1)
High Calorie/High Protein Diet	1 (0.6)	4 (0.2)
Low Fat Diet	0 (0.0)	12 (0.7)
Low Sodium Diet	0 (0.0)	45 (2.7)
Minced Diet	0 (0.0)	1 (0.1)
NPO	2 (1.2)	1 (0.1)
Purine Restricted Diet	0 (0.0)	3 (0.2)
Regular Diet	97 (60.6)	1400 (85.5)
Renal Diet	6 (3.8)	2 (0.1)
Soft Diet	1 (0.6)	1 (0.1)
BMI		*
[15,20)	3 (1.9)	77 (4.7)
[20,25)	37 (23.1)	568 (34.7)
[25,30)	83 (51.9)	672 (41.1)
30+	37 (23.1)	320 (19.5)
Diabetes (Yes (%))	16 (10.0)	68 (4.2) *
Hypertension (Yes (%))	13 (8.1)	72 (4.4)
Asthma (Yes (%))	3 (1.9)	20 (1.2)
CAD (Yes (%))	1 (0.6)	5 (0.3)
Ischemic (Yes (%))	2 (1.2)	10 (0.6)
COPD (Yes (%))	0 (0.0)	1 (0.1)
Cancer (Yes (%))	3 (1.9)	24 (1.5)
CKD (Yes (%))	9 (5.6)	6 (0.4) *
ESRD (Yes (%))	5 (3.1)	2 (0.1) *

*p≤.05, ttest, Mann-Whitney U test, *χ*^2^ test or Fisher’s exact test, as appropriate.,

** GCC: Kuwait, KSA, Baharin, Oman, and Qatar.

**Table 2:** Baseline characteristics statistical analysis: Baseline characteristics of patients stratified by severity measure by accessing the ICU, mean (SD) or N (%).

	ICU	NON-ICU
N	160	1637
Cough (Yes (%))	101 (63.1)	563 (34.4) *
Confusion		*
Alert	155 (96.9)	1621 (99.0)
Comatose	0 (0.0)	3 (0.2)
Stuporous	0 (0.0)	1 (0.1)
Duplicate Order	0 (0.0)	2 (0.1)
Other	5 (3.1)	10 (0.6)
Temperature oral (C)	37.18 (0.63)	36.87 (0.44) *
Oxygen saturation	95.53 (8.30)	98.77 (1.34) *
Modified Early Warning Score (MEWS)		*
0	66 (41.2)	1405 (85.8)
1	34 (21.2)	161 (9.8)
2	22 (13.8)	43 (2.6)
3	17 (10.6)	18 (1.1)
4	12 (7.5)	5 (0.3)
5	6 (3.8)	4 (0.2)
6	1 (0.6)	0 (0.0)
7	1 (0.6)	1 (0.1)
8	1 (0.6)	0 (0.0)
Respiratory rate	21.37 (5.90)	18.11 (2.23)*
Systolic BP	136.80 (17.86)	131.83 (15.72)*
Diastolic BO	82.00 (12.85)	81.21 (11.98)
Bili total	9.76 (12.25)	6.52 (6.42)*
AST	37.76 (26.61)	22.65 (19.55)*
ALT	34.13 (24.53)	27.99 (27.60)*
Platelet	218.71 (80.30)	226.41 (87.59)
Troponin T	7.51 (22.31)	0.41 (2.44)*
Vitamin D OH (level)	1.13 (8.47)	0.05 (2.20)*
EGFR	62.62 (48.33)	18.24 (40.65)*
RBC	4.89 (0.90)	4.88 (1.35)
HGB	134.94 (22.99)	137.49 (37.27)
Hydroxychloroquine (Yes (%))	114 (71.2)	1346 (82.2)*
Tocilizumab (Yes (%))	4 (2.5)	12 (0.7)
Favipiravir (Yes (%))	72 (45.0)	856 (52.3)
Lopinavir ritonavir (Yes (%))	59 (36.9)	232 (14.2)*
Chloroquine (Yes (%))	5 (3.1)	201 (12.3)*
Azithromycin (Yes (%))	30 (18.8)	341 (20.8)

*p≤.05, ttest, Mann-Whitney U test, *χ*^2^ test or Fisher’s exact test, as appropriate

**Table 3:** Baseline characteristics statistical analysis: Baseline characteristics of patients stratified by severity measure by accessing the ICU, mean (SD) or N (%).

	ICU	NON-ICU
N	160	1637
Blood group		
A NEG	3 (1.9)	25 (1.5)
A POS	49 (30.6)	432 (26.4)
AB NEG	0 (0.0)	7 (0.4)
AB POS	8 (5.0)	126 (7.7)
B NEG	3 (1.9)	23 (1.4)
B POS	37 (23.1)	438 (26.8)
O NEG	5 (3.1)	32 (2.0)
O POS	55 (34.4)	554 (33.8)
Amylase (U/L)	76.89 (68.44)	74.84 (37.71)
C reactive protein (mg/L)	67.37 (83.16)	11.62 (37.75)*
Calcium (mg/dL)	2.21 (0.14)	2.34 (0.12)*
Chloride (mEq/L)	99.52 (4.34)	101.29 (2.71)*
CO2 (mEq/L)	23.13 (3.08)	24.82 (2.32)*
Creatinine (mol/L)	113.60 (172.50)	80.55 (32.10)*
Ferritin (ng/ml)	1082.22 (1847.16)	342.49 (424.65)*
Glucose-6-phosphate dehydrogenase (U/g) Hb	7.51 (3.52)	7.82 (3.19)
Glucose random (mg/dL)	7.68 (3.92)	6.22 (2.46)*
LDH (mg/dL)	370.32 (235.87)	220.62 (75.84)*
Lipase (U/L)	57.89 (89.25)	41.62 (36.45)*
Lymphcyte (L)	1.38 (0.73)	2.06 (0.77)*
Magnesium (mmol/L)	0.83 (0.11)	0.85 (0.08) *
MCHC (g/dL)	336.93 (13.96)	337.89 (12.70)
Neutrophils	5.13 (3.51)	3.70 (1.87)*
Phosphorus (mg/dL)	1.06 (0.33)	1.14 (0.23)*
Potassium (mmol/L)	4.00 (0.54)	4.09 (0.39)*
Sodium (mEq/L)	136.98 (4.10)	139.53 (2.62)*
Urea (mmol/L)	5.08 (4.53)	3.80 (1.50)*
Uric acid (mg/L)	274.63 (96.25)	307.61 (90.41)*
WBC (x10 ⌃9 /L)	7.18 (3.83)	6.42 (2.15)*

*p≤.05, ttest, Mann-Whitney U test, *χ*^2^ test or Fisher’s exact test, as appropriate

### Statistical and machine learning models

**Fig 2:**
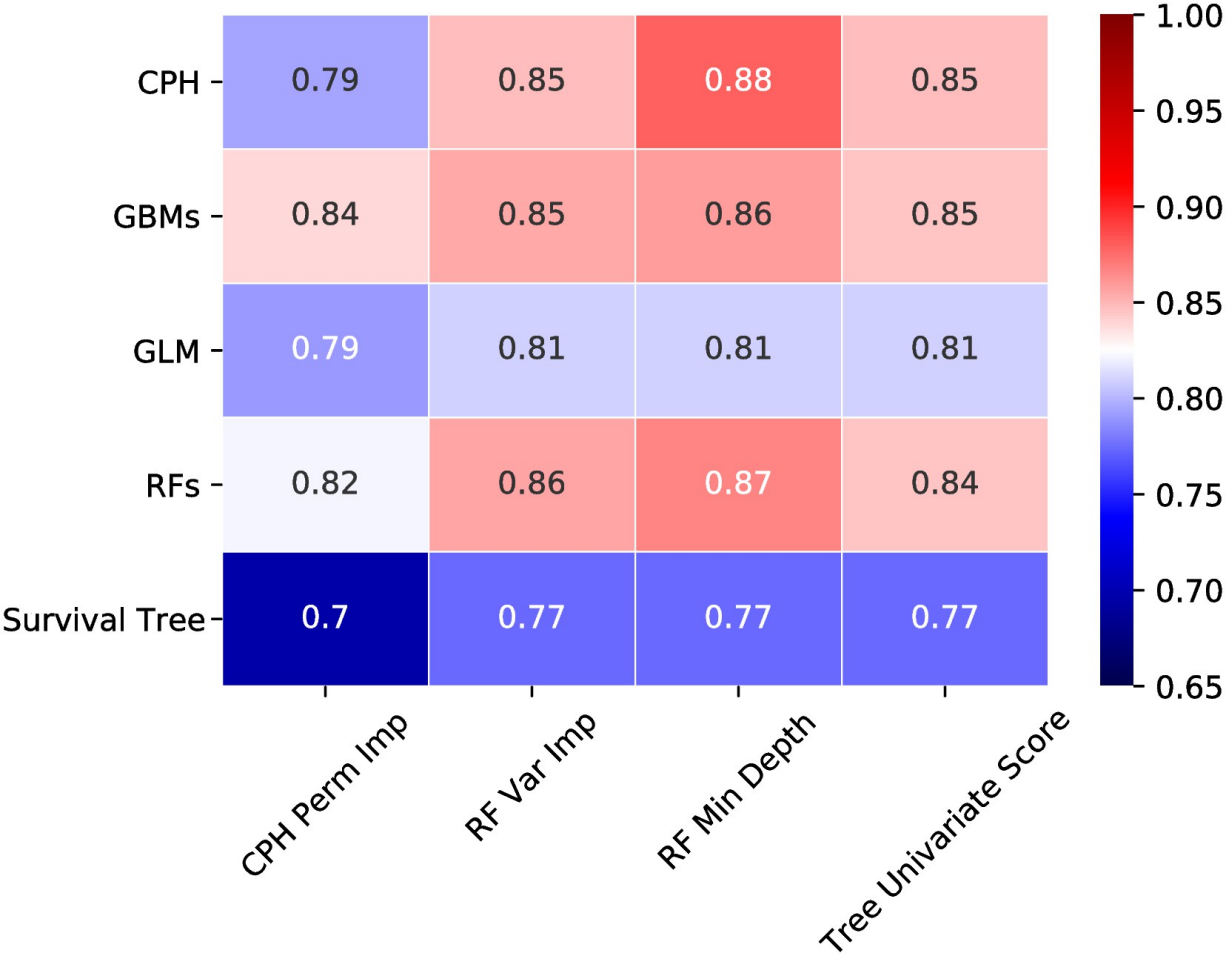
Models hyperparameters tuning: Heatmap of mean C-index (5-fold cross validation) of the best combination of the hyperparameters and feature selections methods; also we tune the threshold for selecting the highest importance features.

Fig 2 illustrates hyperparameters tuning matrix for the combination of different models and feature selection methods. The heatmap shows the C-Index mean value for 5-fold cross validation, across all the models’ random forest minimal depth yield the best selected features. Model chosen hyperparameters and number of features selected for each model reported in Table 4. As it clears in the Table, the percentage of features selected for each model is range from 10 to 49 features. We trained all model with the selected features reported and the completed features (Table 5). The table shows the C-index from the combination of the subset of selected features and the models. Gradient Boosting Machines (GBMs) slightly outperforms CPH and RFs when trained on the feature selected from CPH, GBMs,GLM, RFs and Survival Tree.

**Table 4:** Models’ hyperparameters: Machine learning models parametrized using random search optimization algorithm of 20 different parameter settings with a 5-fold cross validation to maximize the C-index.

Model	Parameter	Parameter Space	Chosen Value	Feature Selection Model (number of selected features)
Multivariate Cox Proportional Hazard	Percentage of selected important feature selected	Numerical space ranging from 0 to 0.9	0.18	RF Min Depth (10)
Survival Tree	Minsplit	{20, 25, 50,75}	20	RF Min Depth (33)
	Maxdepth	{1, 2, 3, 4, 5, 10}	3	
	Cp	numerical space ranging from 0.0 to 1	0.043182235	
	Percentage of selected important feature	numerical space ranging from 0 to 0.9	0.595	
Random Forests	Splitrule	{logrank, logrankscore}	Logrank	RF Min Depth (44)
	No. trees	{25, 50, 75, 100}	75	
	Max. depth	{1, 2, 3, 4, 5, 10}	5	
	Percentage of selected important feature	numerical space ranging from 0 to 0.9	0.8	
Regularized Generalized Linear Model	Alpha	numerical space ranging from 0.0 to 1	0.947775728	RF Min Depth (49)
	Lambda	2⌃x space ranging from -10 to 10	3.519898881	
	Percentage of selected important feature	numerical space ranging from 0 to 0.9	0.88	
Gradient Boosting Machine	No. trees	{25, 50, 75, 100}	75	RF Min Depth (14)
	Max. depth	{1, 2, 3, 4, 5, 10}	10	
	Learning rate	{0.001,0.01,0.1}	0.1	
	Subsamples	{0.2,0.3,0.4, 0.5,0.6, 0.7, 0.8}	0.8	
	Percentage of selected important feature	numerical space ranging from 0 to 0.9	0.28	

**Table 5:** Selected features and models combined performance using repeated 5-fold cross-validation, C-index with 95% confidence interval(95% CI).

	All	CPH	GBMs	GLM	RFs	Survival Tree
CPH	0.84(0.83,0.85)	0.87(0.86,0.87)	0.87(0.86,0.87)	0.84(0.83,0.85)	0.84(0.83,0.85)	0.86(0.85,0.86)
GBMs	0.86(0.86,0.86)	0.87(0.87,0.88)	**0.88(0.87,0.88)**	0.87(0.86,0.87)	0.87(0.86,0.87)	0.87(0.86,0.87)
GLM	0.50(0.50,0.50)	0.50(0.50,0.50)	0.50(0.50,0.50)	0.50(0.50,0.50)	0.50(0.50,0.50)	0.50(0.50,0.50)
RFs	0.86(0.86,0.86)	0.87(0.87,0.88)	0.87(0.87,0.88)	0.86(0.86,0.87)	0.86(0.86,0.87)	0.87(0.86,0.87)
Survival Tree	0.78(0.78,0.79)	0.78(0.78,0.79)	0.78(0.78,0.79)	0.78(0.78,0.79)	0.78(0.78,0.79)	0.78(0.78,0.79)

### Model evaluation and explanation

**Fig 3:**
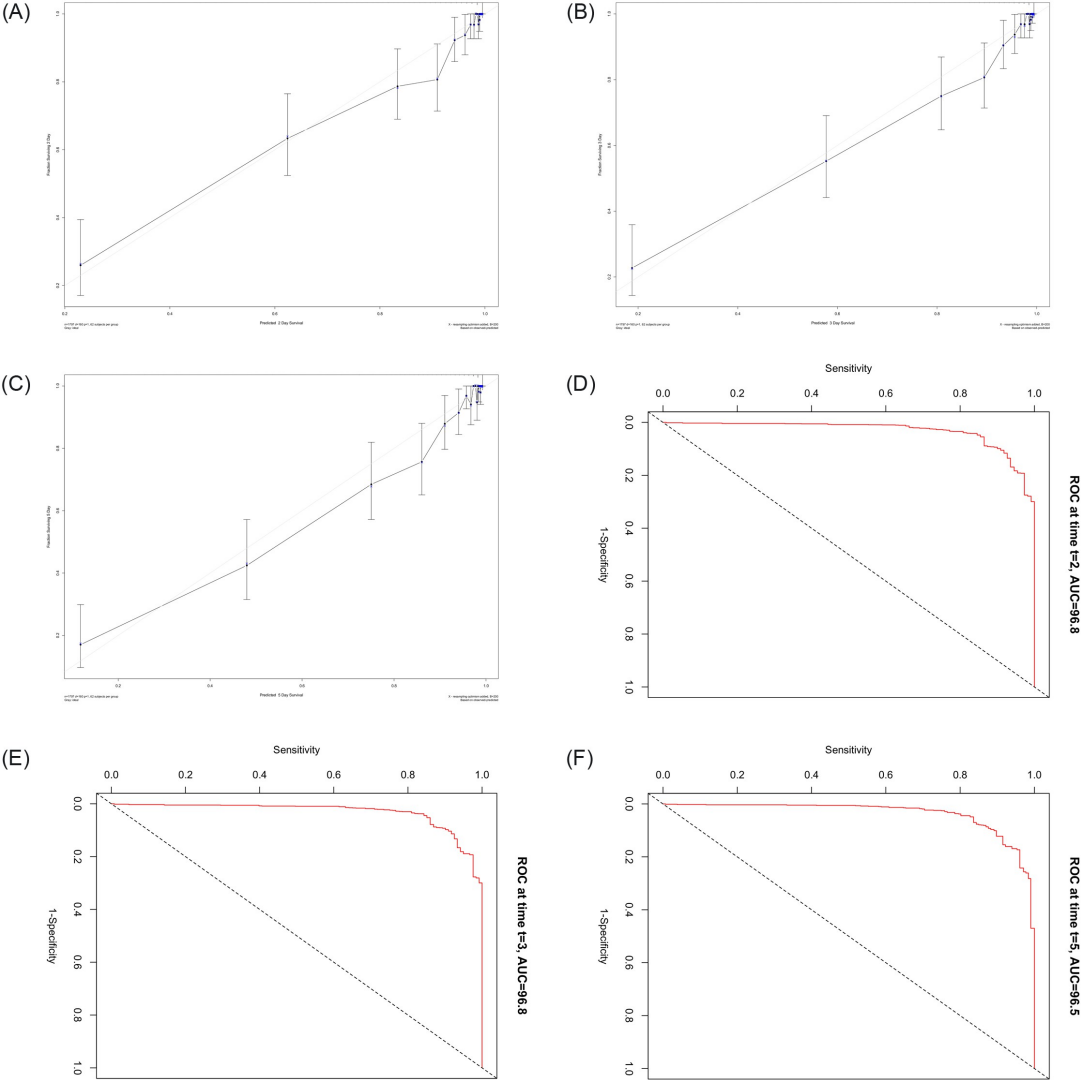
Calibration curves and time-dependent ROC curve of the gradient boosting machine (GBM) model: Calibration curves of predicted compared with observed ICU access after 2 Days (a), 3 Days (b) and 5 Days (c) of hospital administration. Time-dependent ROC curve for the ICU admission predicting after after 2 Days (d),3 Days (e) and 5 Days (f) of hospital administration.

Using Kruskal-Wallis test showed a significant difference between models’ performance (H = 926.50, p ≤ 0.05). Followed by Namanya’s multiple comparisons tests (Table 6); the test showed that there is not a significant difference between GBMs and CPH or RFs performance at 95 significance level. Based on Table 5, we selected the GBM model for further analysis and interpretation. Calibration curves for the probability of 2, 3, and 5 days ICU access showed excellent agreement between model prediction and actual observation (Fig 3a–3c). The Time-dependent ROC curve (discrimination accuracy) for the predicting the ICU access was 96.8%, 96.8%, and 96.5% for 2,3, and 5 days, respectively (Fig 3d–3f).

**Table 6:** Results of Namanya’s post hoc multiple comparisons for different models.

	CPH	GBMs	GLM	RFs	Survival Tree
CPH	1.00				
GBMs	0.49	1.00			
GLM	0.00	0.00	1.00		
RFs	0.75	0.99	0.00	1.00	
Survival Tree	0.12	0.00	0.47	0.00	1.00

* Statistically significant difference: p≤0.05

**Fig 4:**
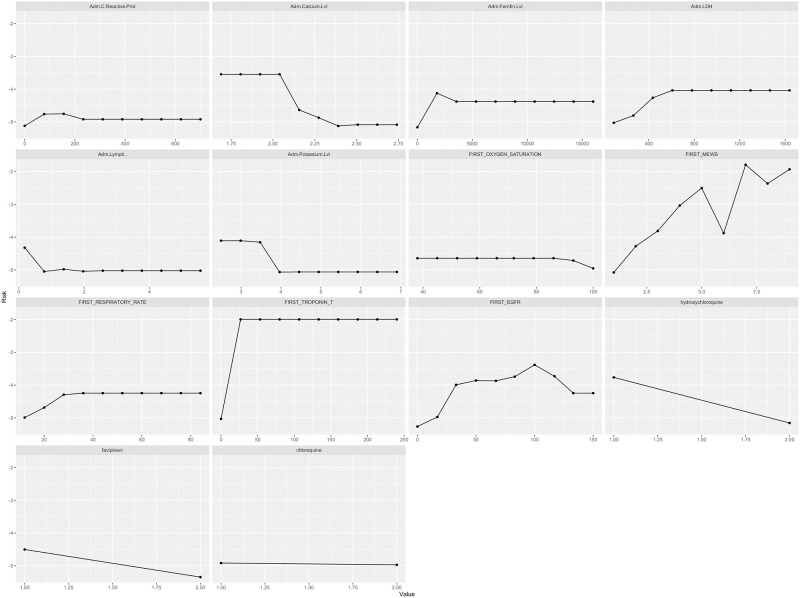
Partial dependence plots inferred from gradient boosting machine (GBM) model using random forest minimal depth features subset: The lines present the change in the risk to access the ICU across selected variable of interest whilst holding other variables constant.

Finally, we explore the model feature of interest marginal risk effect using PDPs. Fig 4 shows the risk of disease severity rises with increase in the C-reactive protein, ferritin level, lactate dehydrogenase level (LDH), Modified Early Warning Score (MEWS), respiratory rate, and troponin levels. In addition, the risk increase with the lower concentrations of potassium, calcium, oxygen saturation and estimated glomerular filtration rate (eGFR) and lymphocytes. Finally, COVID19 adopted treatment region using hydroxychloroquine and favipiravir reduce the severity of the COVID19.

## Discussion

Various studies have been conducted to leverage Machine learning (ML) to characterize the clinical risk factors for COVID-19 severity globally. To the best of our knowledge, this is the first study in the UAE to utilize data from electronic health records (HER) to facilitate clinical decision making during the COVID-19 pandemic using Machine Learning for survival analysis. In our study we identified the risk factors that increase the severity of the COVID-19 (Fig 4) in a UAE cohort. The highest clinical risk variable featured by the model were inflammatory markers including C-reactive protein, Ferritin, and Lactic Dehydrogenase (LDH). Around seventy-six of the cases with elevated CRP upon admission entered ICU. As reported in the literature, elevated level of C-reactive protein might be an indicator of COVID-19 severity and/or mortality [17] and can be used as biomarker to identify patients’ progression status [18]. Elevated LDH has been reported to be in association with respiratory failure in COVID-19 patients. It has been tagged as COVID-19 severe marker with six-fold increase in progressing to severe COVID-19 disease [19]. Another factor identified from our study is serum Ferritin that has been reported as predictor of patient’s severity with COVID-19. A recent study indicated that elevated Ferritin (over 25 percentile) associated with pulmonary involvement [20] that was targeted as biomarker for therapeutic monitoring of Methylprednisolone [21].

In this study, low serum calcium (hypocalcemia) has been predicated as COVID-19 severity risk factor. A recent meta-analysis [22] reported that hypocalcemia was significantly associated with COVID-19 severity, mortality, number of hospitalization days and admission to the ICU. These findings support that serum calcium level can be a prognostic marker for COVID-19 especially at initial assessments. Another study reported that COVID-19 patient with hypocalcemia was more likely to require high oxygen support during hospitalization and to be admitted to ICU [23]. It is still not clear the role of calcium in the pathophysiology SARS-CoV-2.

Low potassium (Hypokalemia) has been identified as risk factor for COVID-19 severity. Many cohort characteristics reported the association of the hypokalemia with increased severity of COVID-19 among affected patients [24]. Another study reported the association of the hypokalemia and COVID-19 pneumonia (Moreno-Pérez et al, 2020). From both studies, it is suggested the presence of the renin-angiotensin disorder. As the SARS-COV-2 binds to ACE2 enhancing in the degradation of the ACE2. Hence, this reduces the counteraction of ACE2 on renin-angiotensin system (RAS). The main challenge is to maintain the potassium level due to the continuous renal potassium loss. Consequently, this can have possible effect on cardiovascular functions, neurohormonal activation and other vital organs such as the lung. Therefore, sever hypokalemia in COVID-19 patients indicate the consideration of mechanical ventilation [25]. In an Italian cohort, hypokalemia was reported in 41% of the hospitalized patients but not associated with ICU admission or mortality [26].

Another risk factor identified in our study, is low count of lymphocyte (lymphopenia). This findings align with various studies reported the association of lymphopenia severity and hospitalization of COVID-19 patients [27–29]. Multiple mechanisms were proposed to explain lymphocytes deficiency in COVID-19 including that the virus directly infect lymphocytes resulting in cell destruction as the lymphocyte express ACE2 receptors on its surface. However, further studies needed to understand the underlying reasoning for lymphopenia being an indicator of for COVID-19 severity and poor outcome.

Low oxygen saturation (Hypoxemia) and high respiratory rate upon admission are other COVID-19 severity risk factors selected in our study. The relative risk factor is higher in patient with oxygen saturation below 88% and respiratory rate below 38 breath per minutes. As reported in literature oxygen saturation below 90% is a predictor risk factor for sever COVID-19 and /or mortality [30]. These factors can provide a clinical indication upon admission to consider patient for appropriate oxygen supplement and timely access to hospital care especially with the limited critical care resources during COVID-19 pandemic. In addition, hypoxia was reported as an independent marker associated with in hospital mortality in COVID-19 patients [30].

Initial Modified Early Warning Score (MEWS) is an important variable to measure the deterioration of patients’ status in hospital-based setting. In our study the MEWS is a factor to predict the risk of ICU admission (Fig 4). The initial MEWS scoring is a significant factor to indicate the ICU admission along especially in patients with silent hypoxemia [31]. The strength of this factor is to leverage the information from HER to provide actionable strategy for COVID-19 patients during pandemic. Hence, this reflects the effect of the clinical decision aided tools on health system.

Elevated cardiac troponin was observed to be a COVID-19 severity predictor risk factor. Cardiac Troponin is well-known myocardial injury marker. Various studies evaluated elevated cardiac troponin as a biomarker of COVID-19 severity and indicative of patient deteteriation [32–34]. In addition, cardiac troponin was evaluated in COVID-19 patients to be a risk factor of severity and an independent predictor of death within 30 days [34]. A recent study evaluated the cut-off of high sensitivity troponin I in non-severe COVID-19 patients as indicator of cardiac damage in the second week of the onset [32].

Low Estimated Glomerular Filtration (eGRF) rate is a risk factor for progression of COVID-19 severity in the studied cohorts. This risk factor has been reported in various studies as a predictor for COVID-19 prognosis [35]. Uribarri et al. [36] clearly demonstrated the impact of renal function from an international HOPE COVID-19 (Health Outcome Predictive Evaluation for COVID-19) Registry. Upon admission of COVID-19 patients, kidney dysfunction is common with various possible complication such as renal failure or in-hospital mortality [35]. Furthermore, patients with eGFR below 60ml/min/m2 were found to have higher risk worse prognosis because of respiratory failure and sepsis. Fifty six percent of COVID-19 patients with eGFR below 30 upon admission exhibited significant deterioration in their renal function. Renal involvement in COVID-19 patients is very important risk factor that requires critical follow up during hospitalization to avoid potential renal complication. Furthermore, early identification of kidney injury can predict COVID-19 progression and poor prognosis [37].

In this study model we have considered the COVID-19 treatment part of the analysis since we are evaluating the risk factor during the hospitalization for ICU admission. As part of the initial treatment offered for inpatients with COVID-19 during early pandemic episode, Hydroxychrolquine and other antiviral therapy such as Favipiravir were offered. In Fig 4, two pharmacotherapies were identified as a supported factor to avoid admission to ICU. The findings from this model do not evaluate the treatment effectiveness independently as most of the cohort patients were under different pharmacotherapies (n = 735). However, the results only evaluate the Hydroxychrolquine and Favipiravir effect on the ICU admission of COVID-19 patients during hospitalization. Early in the pandemic, with the increasing number of the critically ill patients and desperation of clinician Hydroxychrolquine preliminary data on the March 16, 2020 provided hope as a potential treatment for COVID-D patients across the globe. On the March 28, 2020 US Food and Drug administration (FDA) authorized the early use of Hydroxychrolquine. The data stet in this study represent the initial pandemic stage were most of the symptomatic COVID-19 inpatients received Hydroxychrolquine with or without antiviral pharmacotherapy. Many follow up studies followed reported inconclusive efficacy of Hydroxychloroquine in COVID-19 patients [38–40]. Based on the well-defined scientific evidence, in June 15, 2020 the FDA revoked the approval [41, 42]. In addition, the Hydroxychrolquine is no longer recommended in the UAE.

Favipiravir is an antiviral therapy that was initially used to treat COVID-19 in the early wave of the pandemic. No statistical significance was reported of Favipiravir in relation to oxygen supplement, ICU admission and mortality [43]. Meta analysis reported the lack of Favipiravir effectiveness in reducing mortality among mild to moderate COVID-19 patients [43]. Other studies reported that Favipiravir can trigger viral clearance by 7 days and enhance clinical outcome within 14 days. However, this study recommended further evaluation of the dosing and duration of the treatment to validate the findings [44]. In our study, Favipiravir is identified as a factor that can reduce the chance of ICU admission. However, this finding can be marginal (p-value = 0.068) due to the small sample size in this category in this cohort. Various limitations were encountered while analyzing the low numbered dataset of the ICU group for which full spectrum evaluation was not possible. There are other predictors and features that we did not consider in the implemented models such as lifestyle, viral variant strains, viral load, and severity score. Furthermore, the model was not adjusted for smoking as most of the values were missing from the data set. As most patients in this cohort had pre-existing comorbidities, these patients were on different medications that were not included in this investigation. Medication effectiveness was not part of the analysis as the sample size of each pharmacotherapy groups was under-representative.

Further validation is required to evaluate the performance of the model. Various attributes can be changed due to various factors including the emergence of new virus strains, improvement of management practice, new pharmacotherapy, and vaccination availability around the globe.

## Conclusion

In conclusion, predicting COVID-19 severity and evaluating the risk factors during hospitalization is challenging. However, ML models can assist clinicians in identifying high-risk patients upon admission. The focus of our study is to evaluate the risk factors criteria in UAE COVID-19 cohort to facilitate clinician’s decision on ICU admission at limited critical care resources in fast revolving COVID-19 waves. The findings of our study represent the first study of risk factors from EHR in the UAE at the early pandemic stage. Various clinical marker can be used as a predictor variable for ICU admission including C-reactive protein, ferritin level, lactate dehydrogenase level (LDH), Modified Early Warning Score (MEWS), respiratory rate, troponin levels, and the risk increases with lower potassium level, oxygen saturation and estimated glomerular filtration rate (eGFR). By using the identified features, clinicians can provide altered treatment plans and prioritize ICU admission for high-risk patients. Mortality prediction was not investigated in this study.

## Supporting information

S1 Fig Sociodemographic information missing values. The figure illustrates the number of missing instances in each variable. The variable with more than 70% missing information was excluded from this analysis.
(PDF)

S2 Fig Comorbidity missing values. The figure illustrates the number of missing instances in each variable. The variable with more than 70% missing information was excluded from this analysis.
(PDF)

S3 Fig Symptoms upon admission missing values. The figure illustrates the number of missing instances in each variable. The variable with more than 70% missing information was excluded from this analysis.
(PDF)

S4 Fig Laboratory results upon admission missing values. The figure illustrates the number of missing instances in each variable. The variable with more than 70% missing information was excluded from this analysis.
(PDF)

S5 Fig Vital information upon admission missing values. The figure illustrates the number of missing instances in each variable. The variable with more than 70% missing information was excluded from this analysis.
(PDF)

S6 Fig COVID19 therapy missing values. The figure illustrates the number of missing instances in each variable. The variable with more than 70% missing information was excluded from this analysis.
(PDF)

S7 Fig Missing data patterns in multivariate data. Explore patterns of missingness between levels of included variables. The pairs plots show relationships between missing values (gray) and observed values (Blue) for all the features. The distributions are used to visualize the continuous features, and the proportions are shown for categorical variables.
(PDF)

S8 Fig Missing data patterns in multivariate data. Explore patterns of missingness between levels of included variables. The pairs plots show relationships between missing values (gray) and observed values (Blue) for all the features. The distributions are used to visualize the continuous features, and the proportions are shown for categorical variables (continue).
(PDF)

S9 Fig Missing data patterns in multivariate data. Explore patterns of missingness between levels of included variables. The pairs plots show relationships between missing values (gray) and observed values (Blue) for all the features. The distributions are used to visualize the continuous features, and the proportions are shown for categorical variables (continue).
(PDF)

S10 Fig Missing data patterns in multivariate data. Explore patterns of missingness between levels of included variables. The pairs plots show relationships between missing values (gray) and observed values (Blue) for all the features. The distributions are used to visualize the continuous features, and the proportions are shown for categorical variables (continue).
(PDF)

S11 Fig Missing data patterns in multivariate data. Explore patterns of missingness between levels of included variables. The pairs plots show relationships between missing values (gray) and observed values (Blue) for all the features. The distributions are used to visualize the continuous features, and the proportions are shown for categorical variables (continue).
(PDF)

S12 Fig Missing data patterns in multivariate data. Explore patterns of missingness between levels of included variables. The pairs plots show relationships between missing values (gray) and observed values (Blue) for all the features. The distributions are used to visualize the continuous features, and the proportions are shown for categorical variables (continue).
(PDF)

S13 Fig Missing data patterns in multivariate data. Explore patterns of missingness between levels of included variables. The pairs plots show relationships between missing values (gray) and observed values (Blue) for all the features. The distributions are used to visualize the continuous features, and the proportions are shown for categorical variables (continue).
(PDF)

S14 Fig Missing data patterns in multivariate data. Explore patterns of missingness between levels of included variables. The pairs plots show relationships between missing values (gray) and observed values (Blue) for all the features. The distributions are used to visualize the continuous features, and the proportions are shown for categorical variables (continue).
(PDF)

S15 Fig Missing data patterns in multivariate data. Explore patterns of missingness between levels of included variables. The pairs plots show relationships between missing values (gray) and observed values (Blue) for all the features. The distributions are used to visualize the continuous features, and the proportions are shown for categorical variables (continue).
(PDF)

S16 Fig Missing data patterns in multivariate data. Explore patterns of missingness between levels of included variables. The pairs plots show relationships between missing values (gray) and observed values (Blue) for all the features. The distributions are used to visualize the continuous features, and the proportions are shown for categorical variables (continue).
(PDF)

S17 Fig Missing data patterns in multivariate data. Explore patterns of missingness between levels of included variables. The pairs plots show relationships between missing values (gray) and observed values (Blue) for all the features. The distributions are used to visualize the continuous features, and the proportions are shown for categorical variables (continue).
(PDF)

S18 Fig Missing value imputation using random forest. The figure compare the distribution of the original and imputed data. The magenta points represent the imputed points, and the blue ones show the observed ones. The plots infer that the imputed values are plausible values for the missing points.
(PDF)

S19 Fig Correlation plot. Heat map to visualize the correlation between the study features after removing the highly correlated features (〉0.7 and 〈-0.7).
(PDF)

S1 File (TEX)
