# Supplementary figures and images for: Utilizing machine learning for survival analysis to identify risk factors for COVID-19 intensive care unit admission: A retrospective cohort study from the United Arab Emirates
Source: PLoS One. 2024 Jan 11;19(1):e0291373. doi: 10.1371/journal.pone.0291373 (PMC10783720; doi:10.1371/journal.pone.0291373)

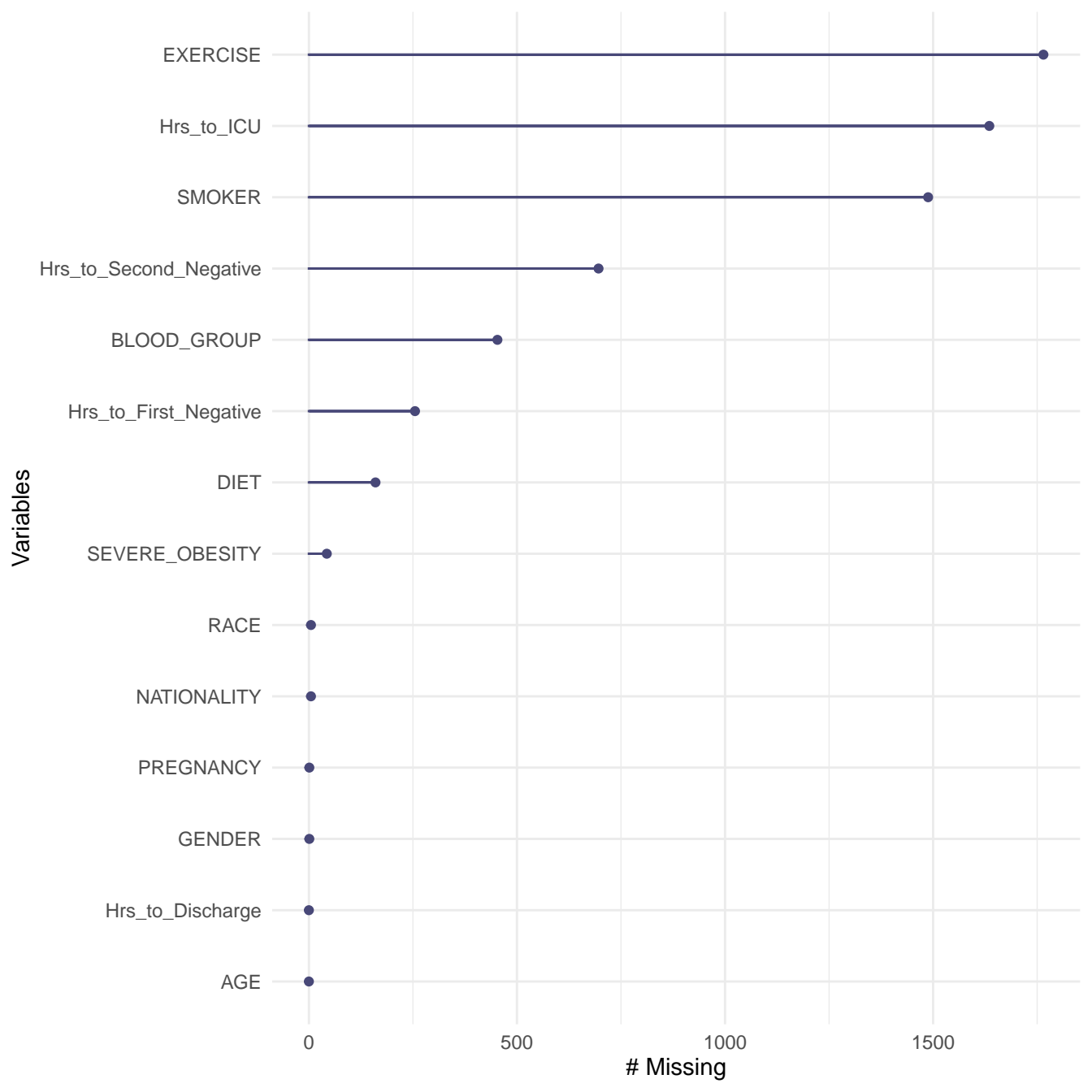

Supplement: S1 Fig — The figure illustrates the number of missing instances in each variable. The variable with more than 70% missing information was excluded from this analysis. (PDF) [file pone.0291373.s001.pdf]

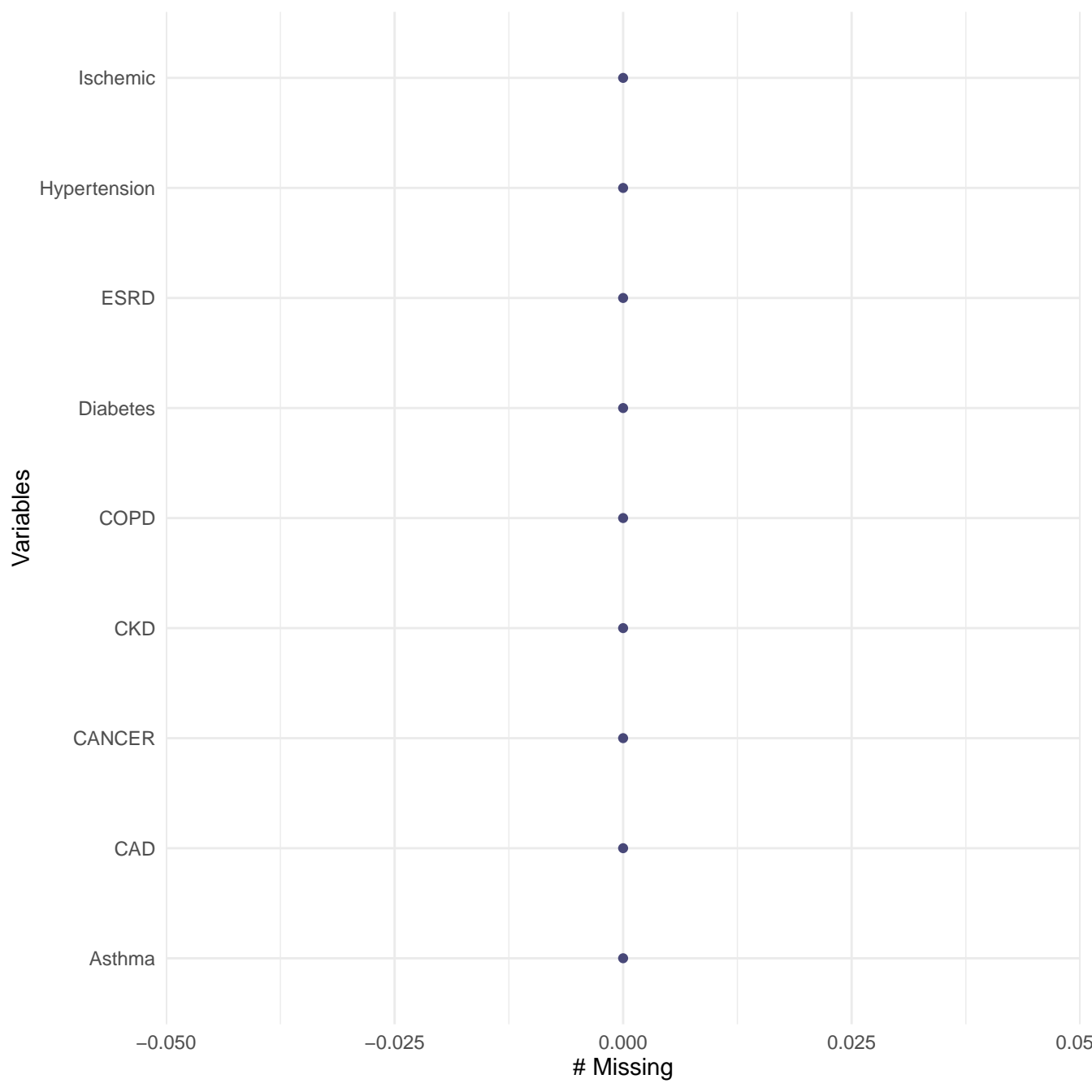

Supplement: S2 Fig — The figure illustrates the number of missing instances in each variable. The variable with more than 70% missing information was excluded from this analysis. (PDF) [file pone.0291373.s002.pdf]

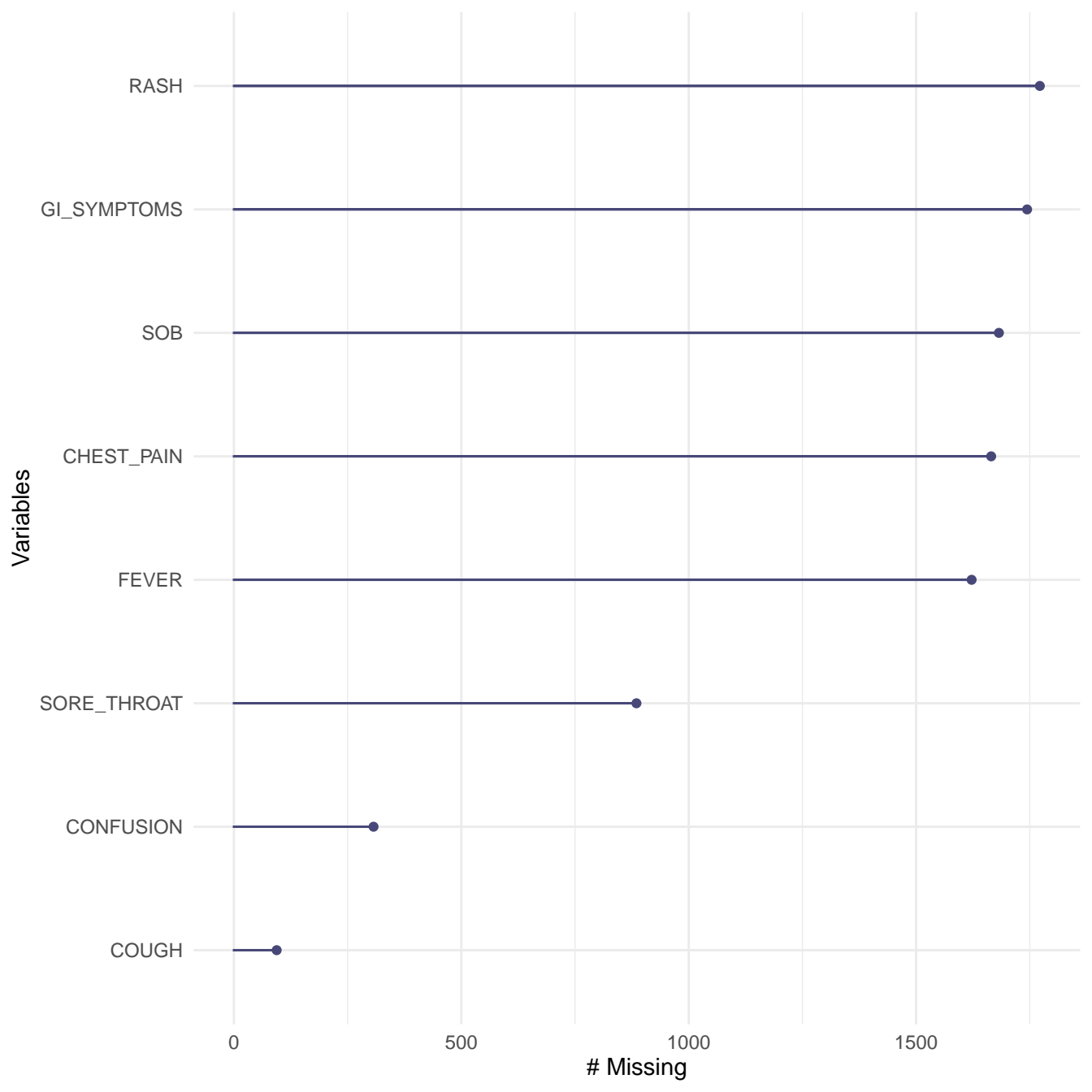

Supplement: S3 Fig — The figure illustrates the number of missing instances in each variable. The variable with more than 70% missing information was excluded from this analysis. (PDF) [file pone.0291373.s003.pdf]

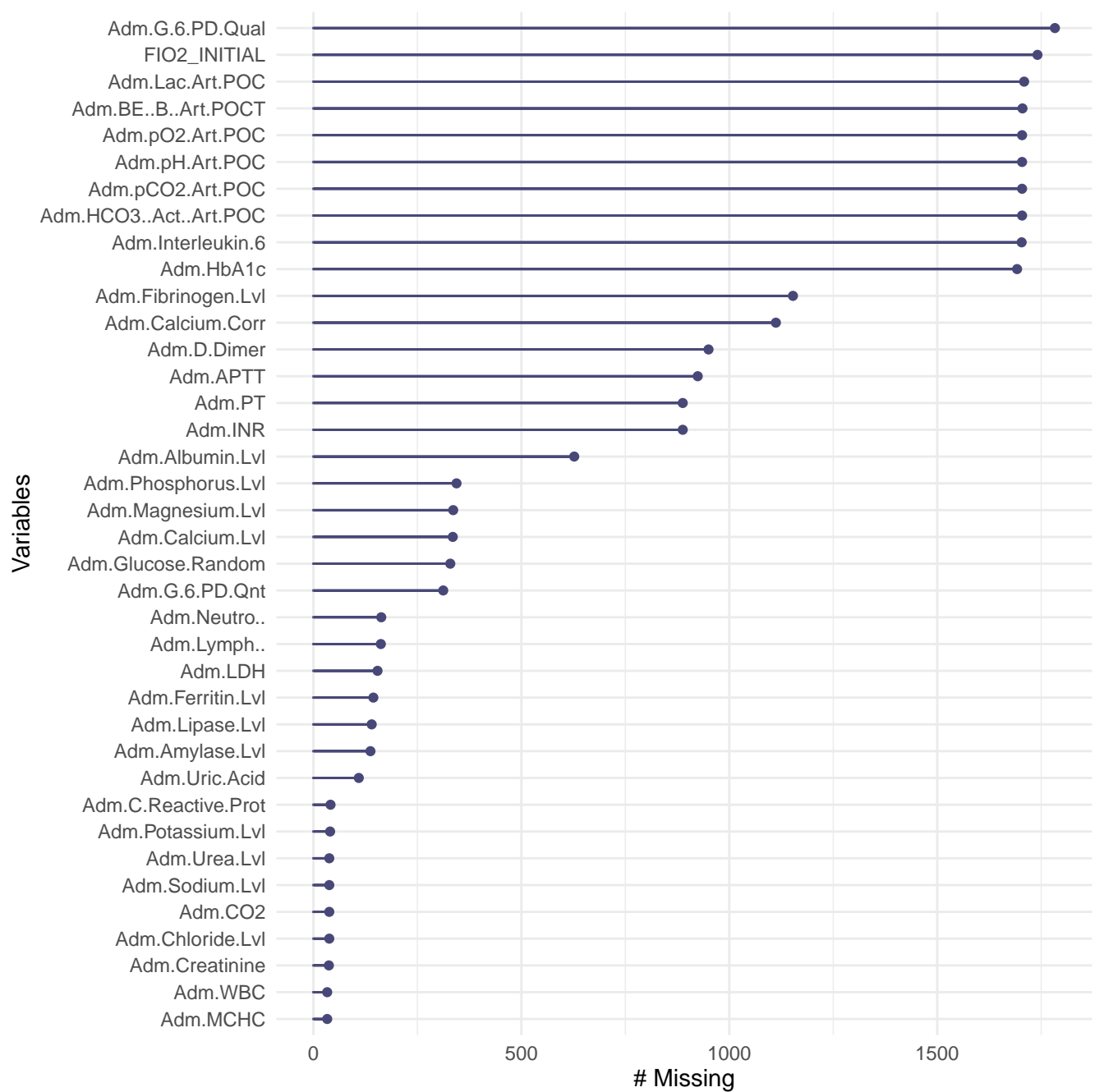

Supplement: S4 Fig — The figure illustrates the number of missing instances in each variable. The variable with more than 70% missing information was excluded from this analysis. (PDF) [file pone.0291373.s004.pdf]

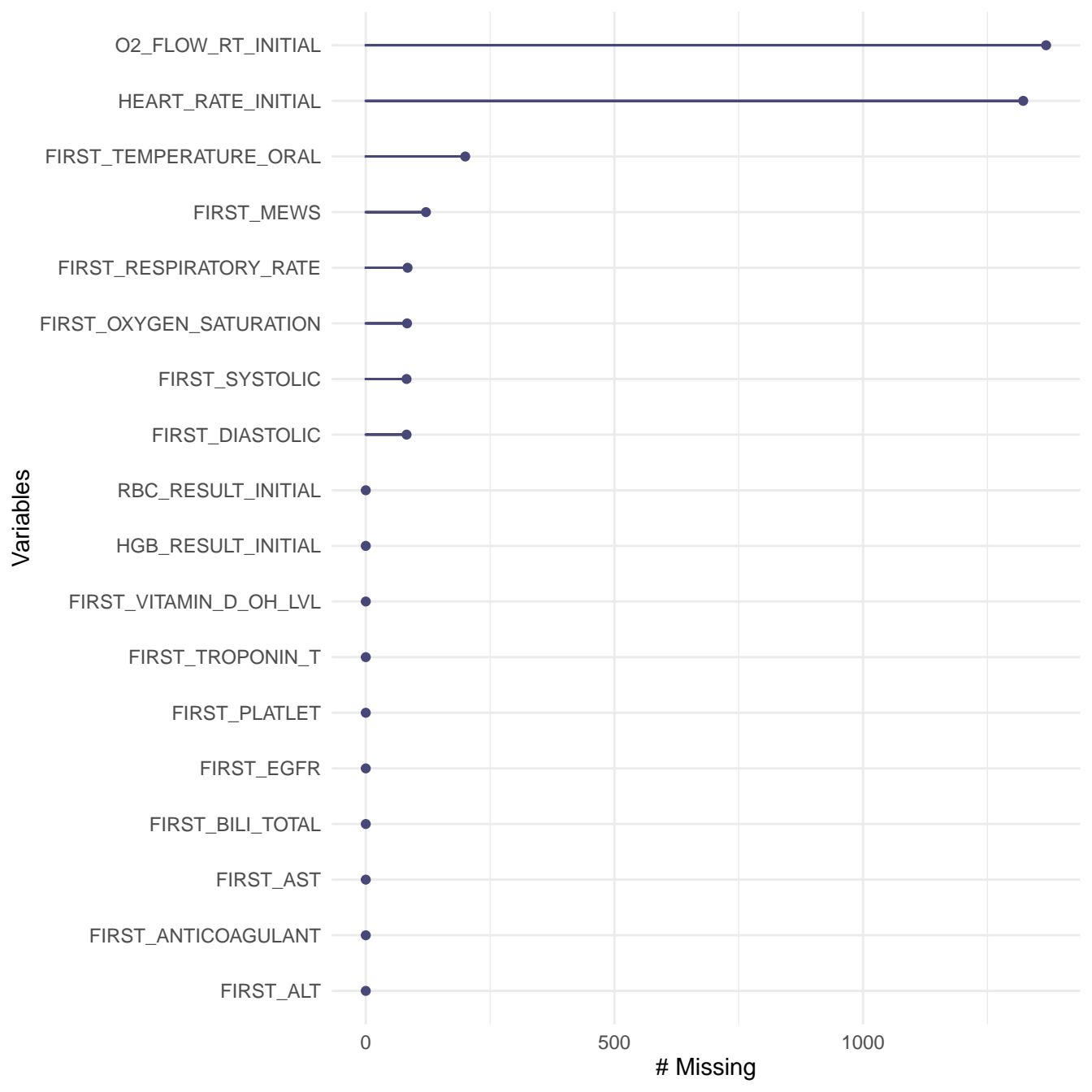

Supplement: S5 Fig — The figure illustrates the number of missing instances in each variable. The variable with more than 70% missing information was excluded from this analysis. (PDF) [file pone.0291373.s005.pdf]

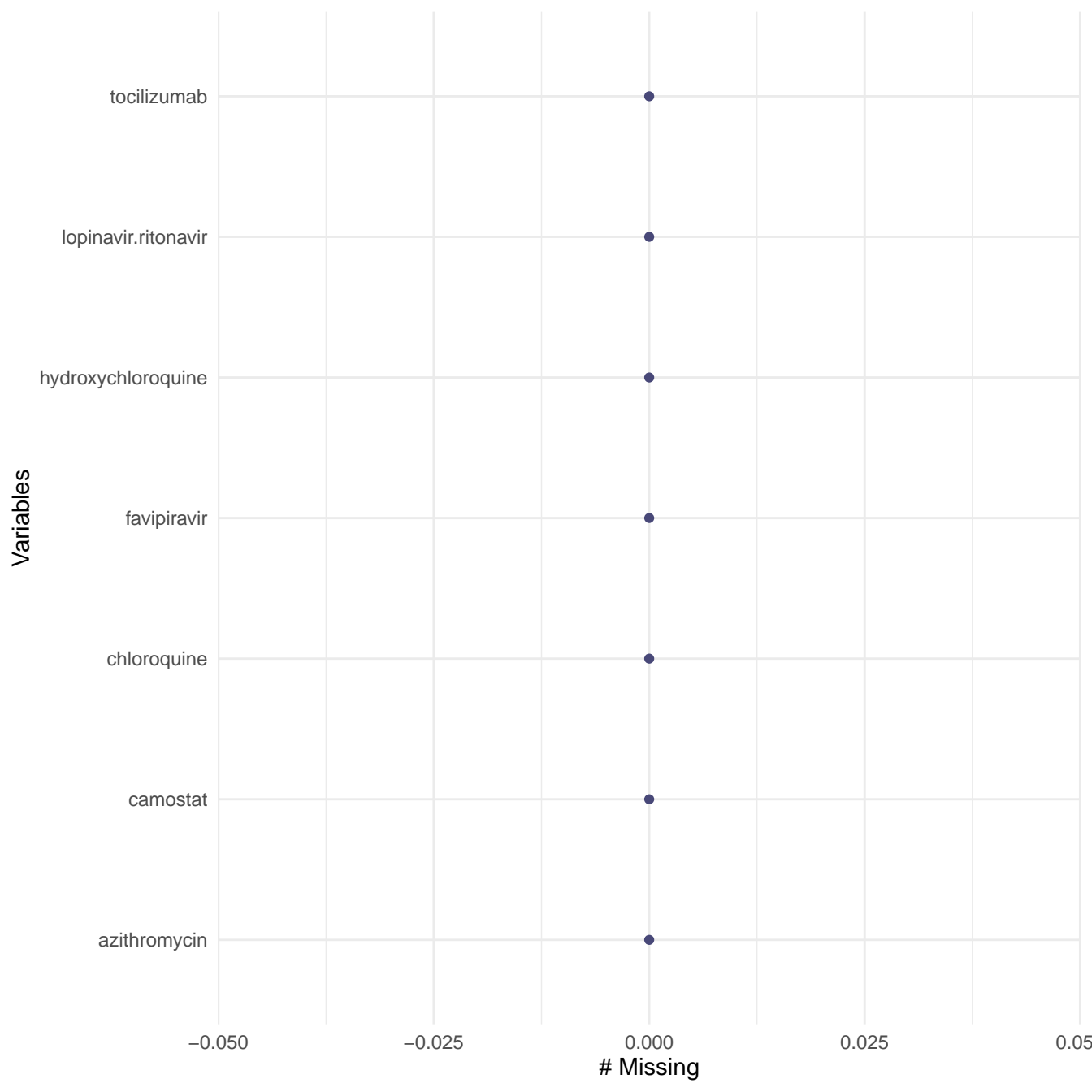

Supplement: S6 Fig — The figure illustrates the number of missing instances in each variable. The variable with more than 70% missing information was excluded from this analysis. (PDF) [file pone.0291373.s006.pdf]

Missing data matrix

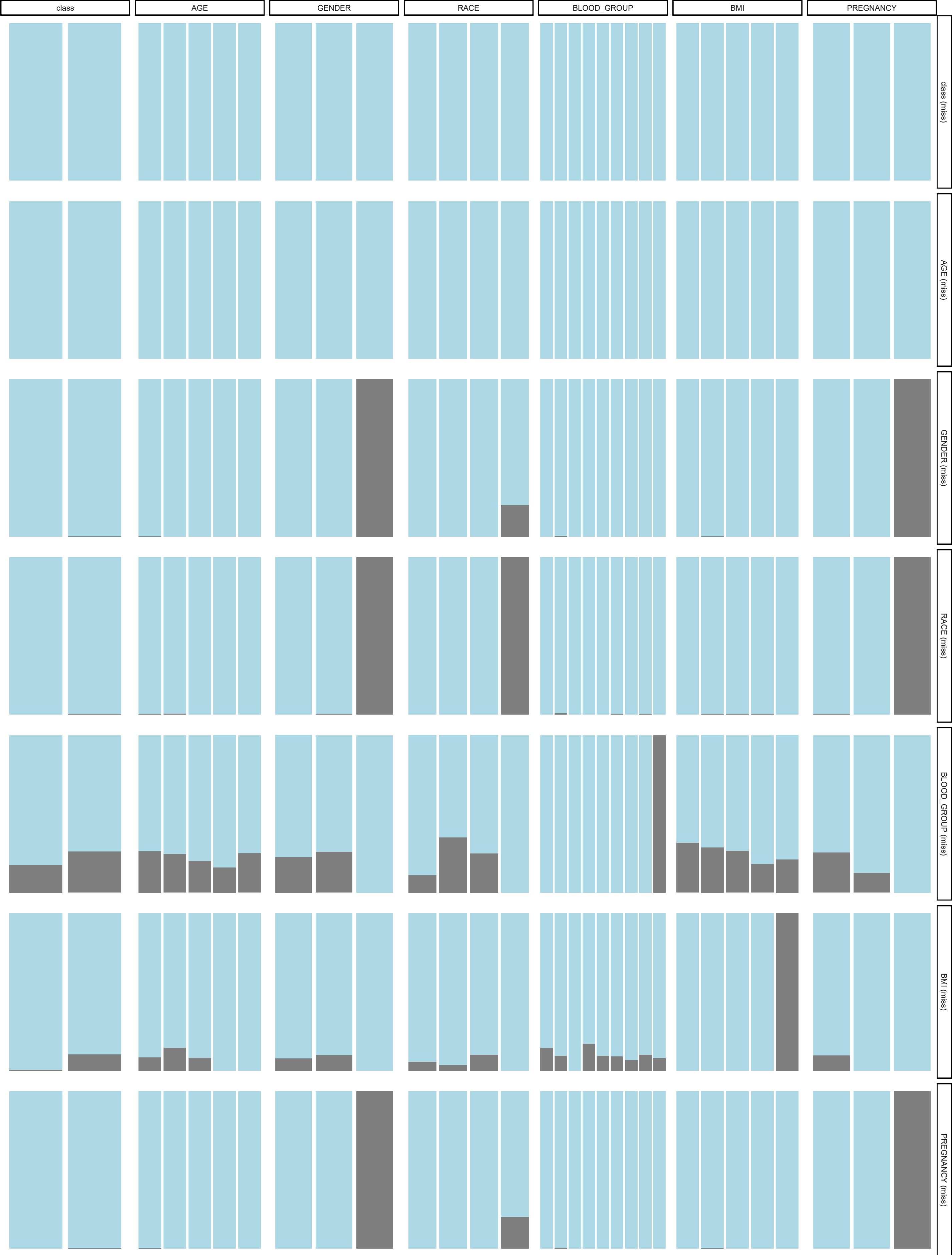

Supplement: S7 Fig — Explore patterns of missingness between levels of included variables. The pairs plots show relationships between missing values (gray) and observed values (Blue) for all the features. The distributions are used to visualize the continuous features, and the proportions are shown for categorical variables. (PDF) [file pone.0291373.s007.pdf]

Missing data matrix

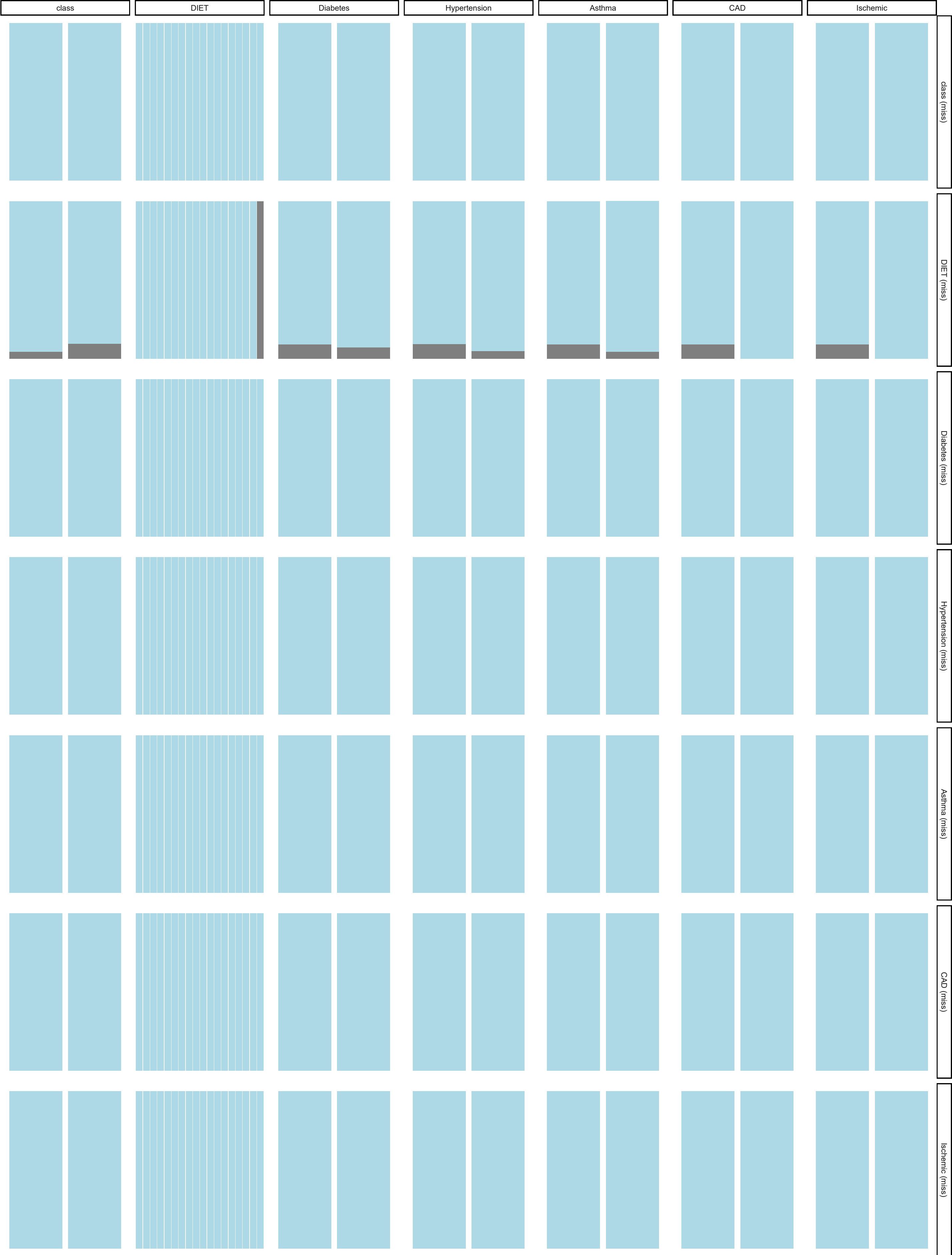

Supplement: S8 Fig — Explore patterns of missingness between levels of included variables. The pairs plots show relationships between missing values (gray) and observed values (Blue) for all the features. The distributions are used to visualize the continuous features, and the proportions are shown for categorical variables (continue). (PDF) [file pone.0291373.s008.pdf]

Missing data matrix

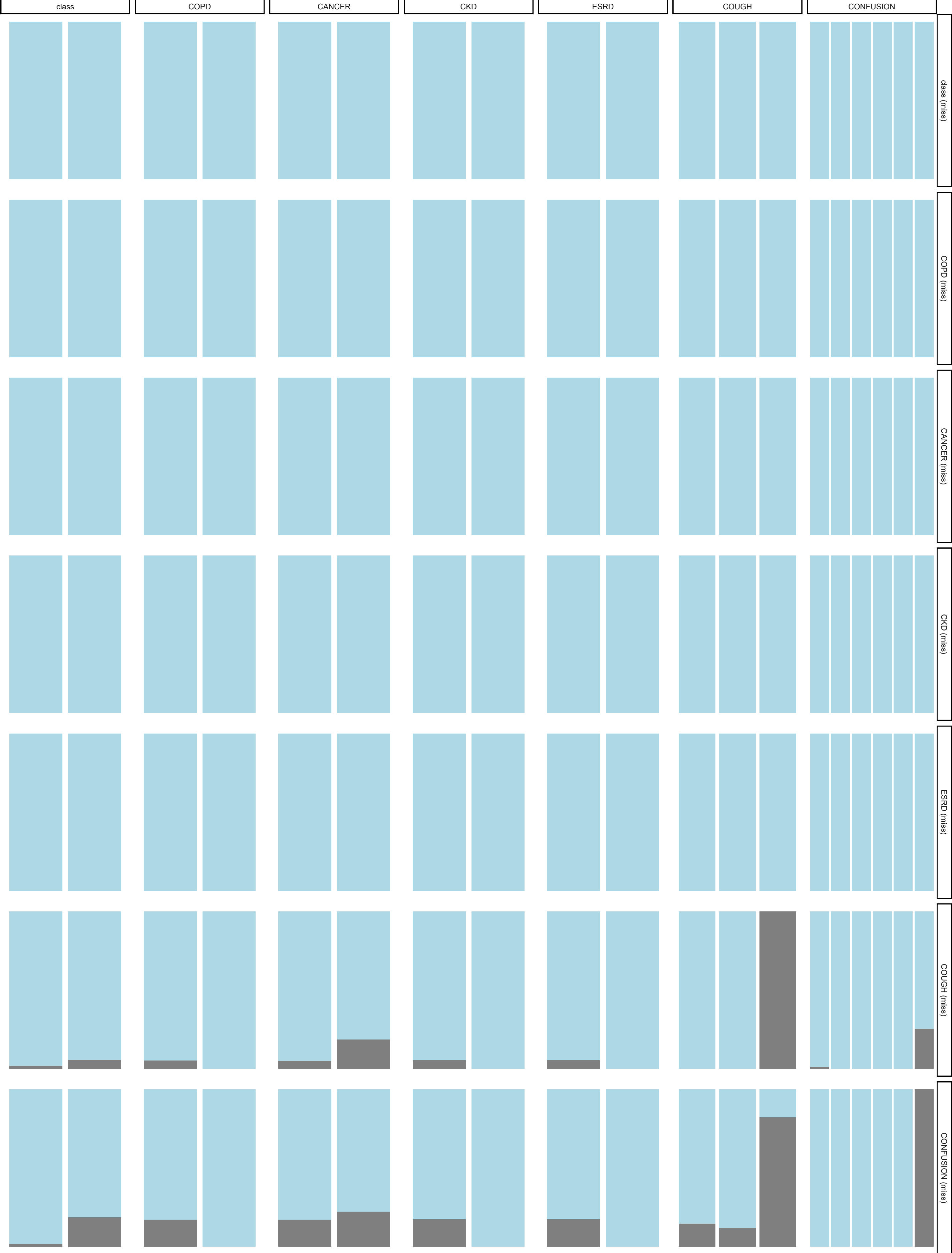

Supplement: S9 Fig — Explore patterns of missingness between levels of included variables. The pairs plots show relationships between missing values (gray) and observed values (Blue) for all the features. The distributions are used to visualize the continuous features, and the proportions are shown for categorical variables (continue). (PDF) [file pone.0291373.s009.pdf]

Missing data matrix

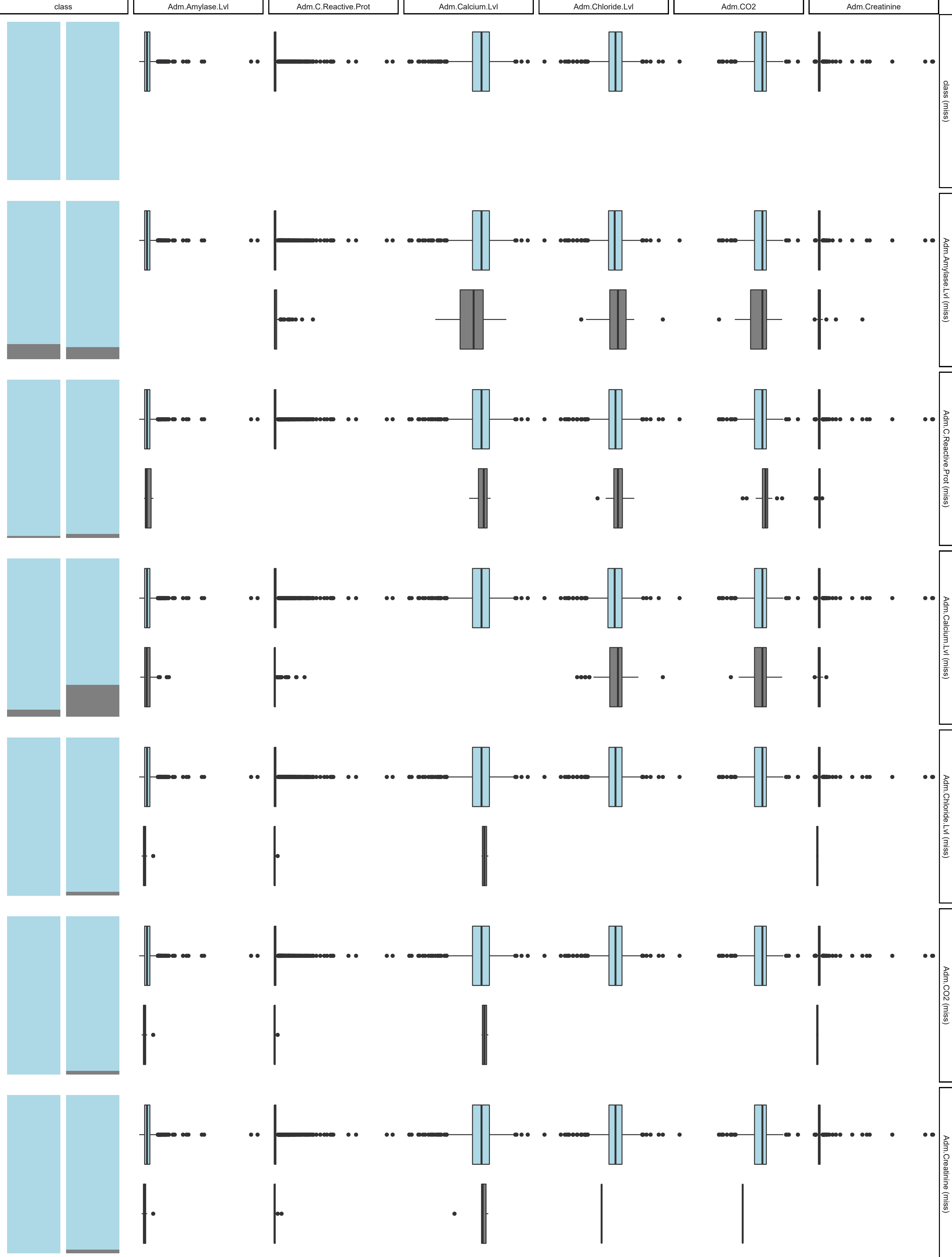

Supplement: S10 Fig — Explore patterns of missingness between levels of included variables. The pairs plots show relationships between missing values (gray) and observed values (Blue) for all the features. The distributions are used to visualize the continuous features, and the proportions are shown for categorical variables (continue). (PDF) [file pone.0291373.s010.pdf]

## Missing data matrix

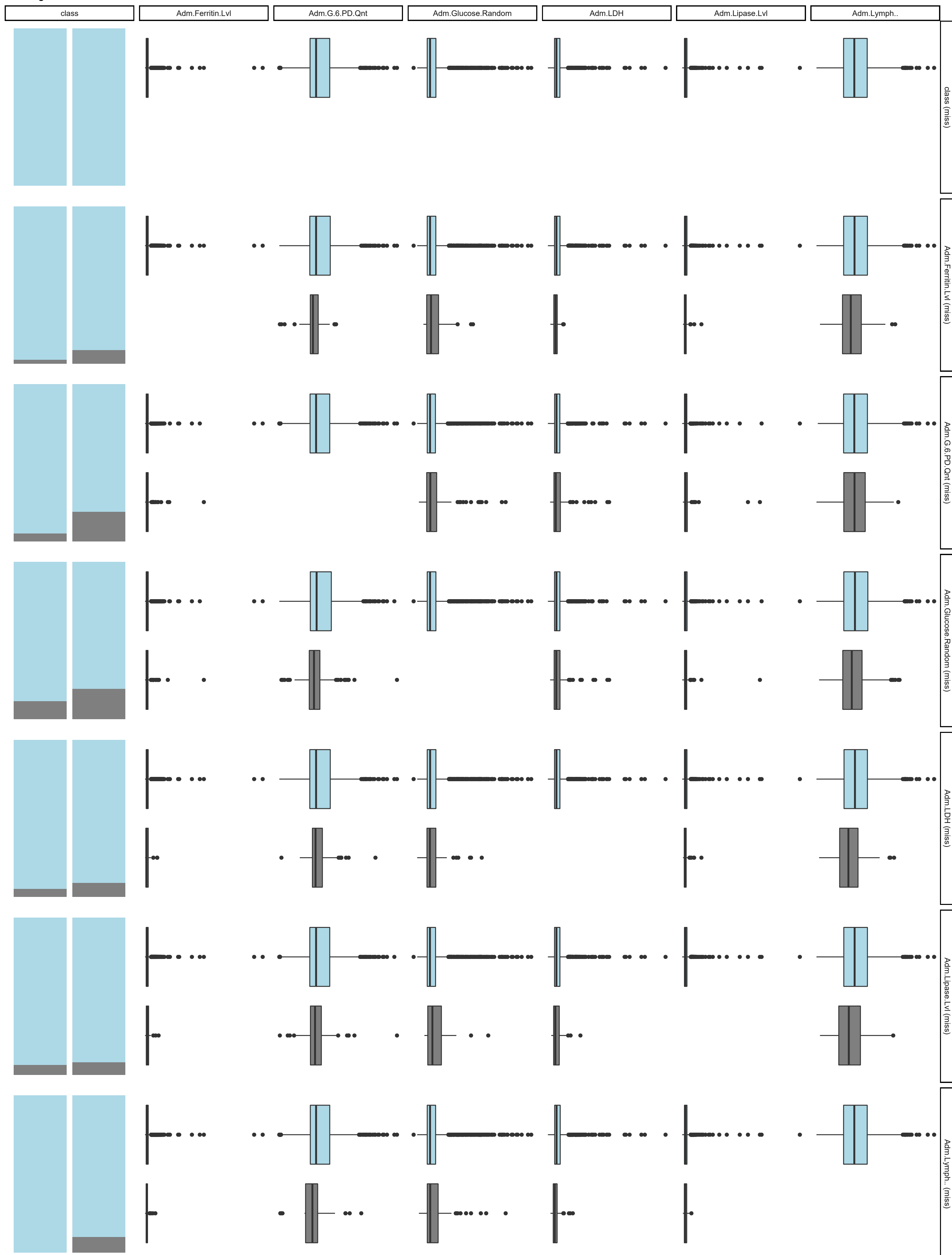

Supplement: S11 Fig — Explore patterns of missingness between levels of included variables. The pairs plots show relationships between missing values (gray) and observed values (Blue) for all the features. The distributions are used to visualize the continuous features, and the proportions are shown for categorical variables (continue). (PDF) [file pone.0291373.s011.pdf]

Missing data matrix

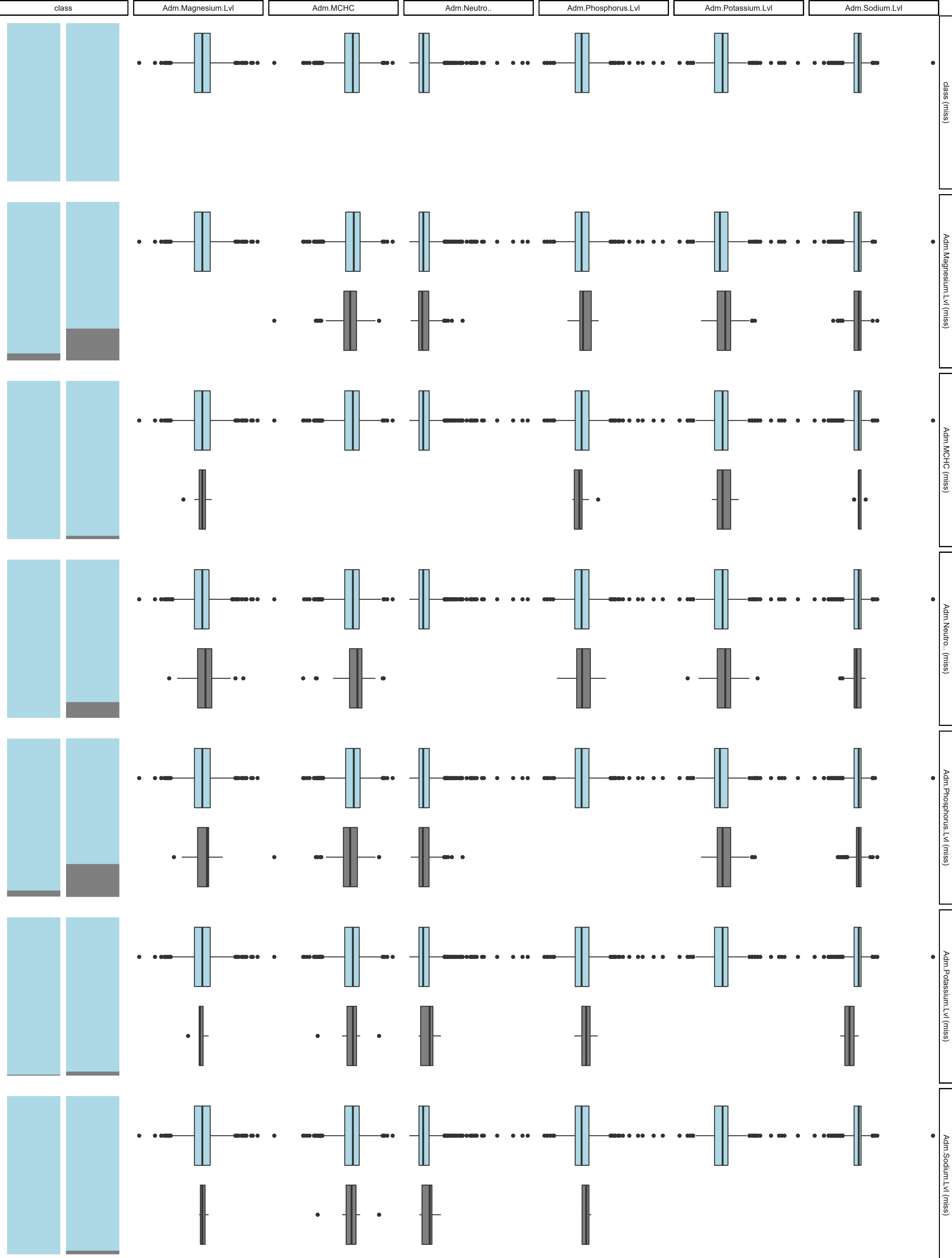

Supplement: S12 Fig — Explore patterns of missingness between levels of included variables. The pairs plots show relationships between missing values (gray) and observed values (Blue) for all the features. The distributions are used to visualize the continuous features, and the proportions are shown for categorical variables (continue). (PDF) [file pone.0291373.s012.pdf]

Missing data matrix

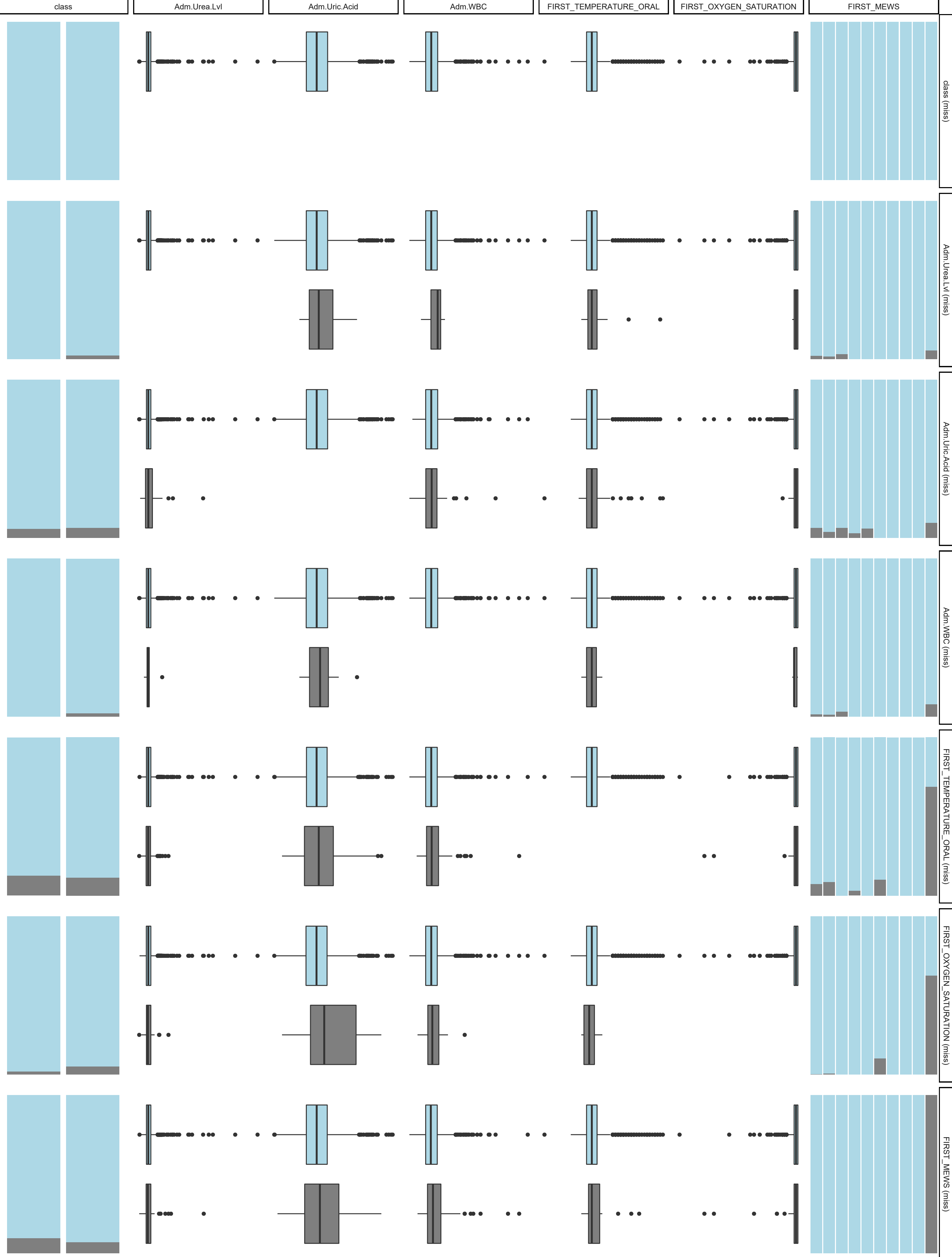

Supplement: S13 Fig — Explore patterns of missingness between levels of included variables. The pairs plots show relationships between missing values (gray) and observed values (Blue) for all the features. The distributions are used to visualize the continuous features, and the proportions are shown for categorical variables (continue). (PDF) [file pone.0291373.s013.pdf]

Missing data matrix

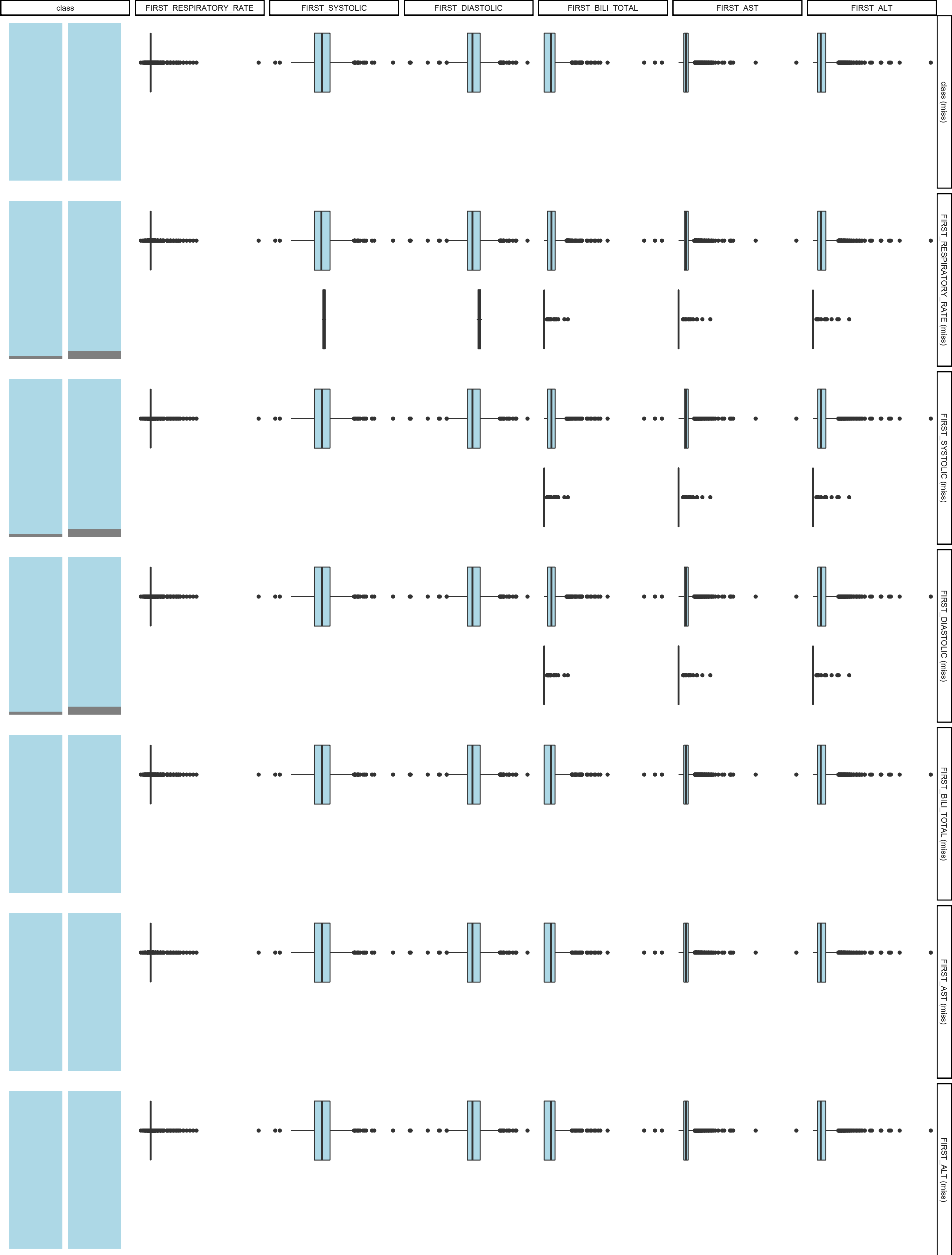

Supplement: S14 Fig — Explore patterns of missingness between levels of included variables. The pairs plots show relationships between missing values (gray) and observed values (Blue) for all the features. The distributions are used to visualize the continuous features, and the proportions are shown for categorical variables (continue). (PDF) [file pone.0291373.s014.pdf]

Missing data matrix

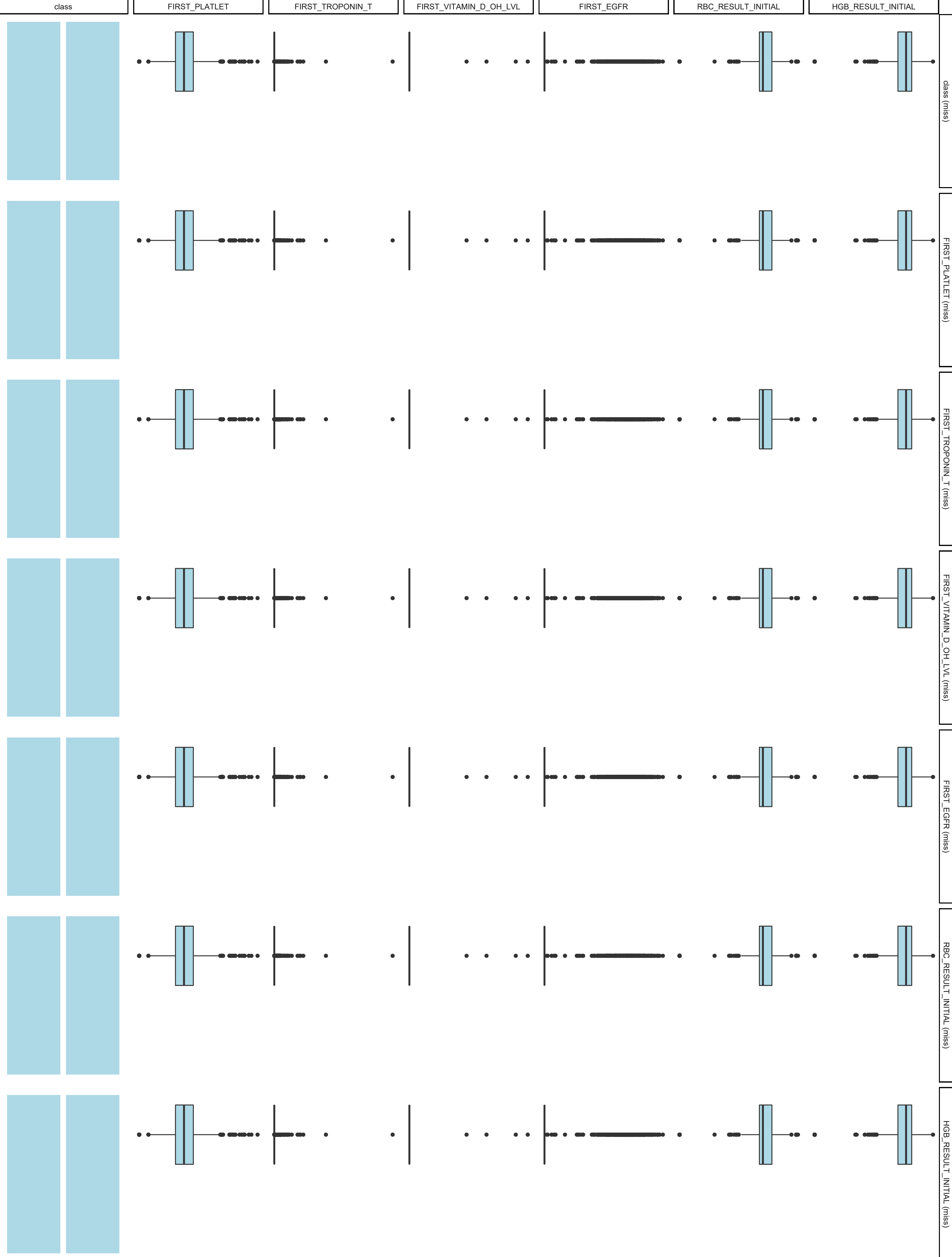

Supplement: S15 Fig — Explore patterns of missingness between levels of included variables. The pairs plots show relationships between missing values (gray) and observed values (Blue) for all the features. The distributions are used to visualize the continuous features, and the proportions are shown for categorical variables (continue). (PDF) [file pone.0291373.s015.pdf]

Missing data matrix

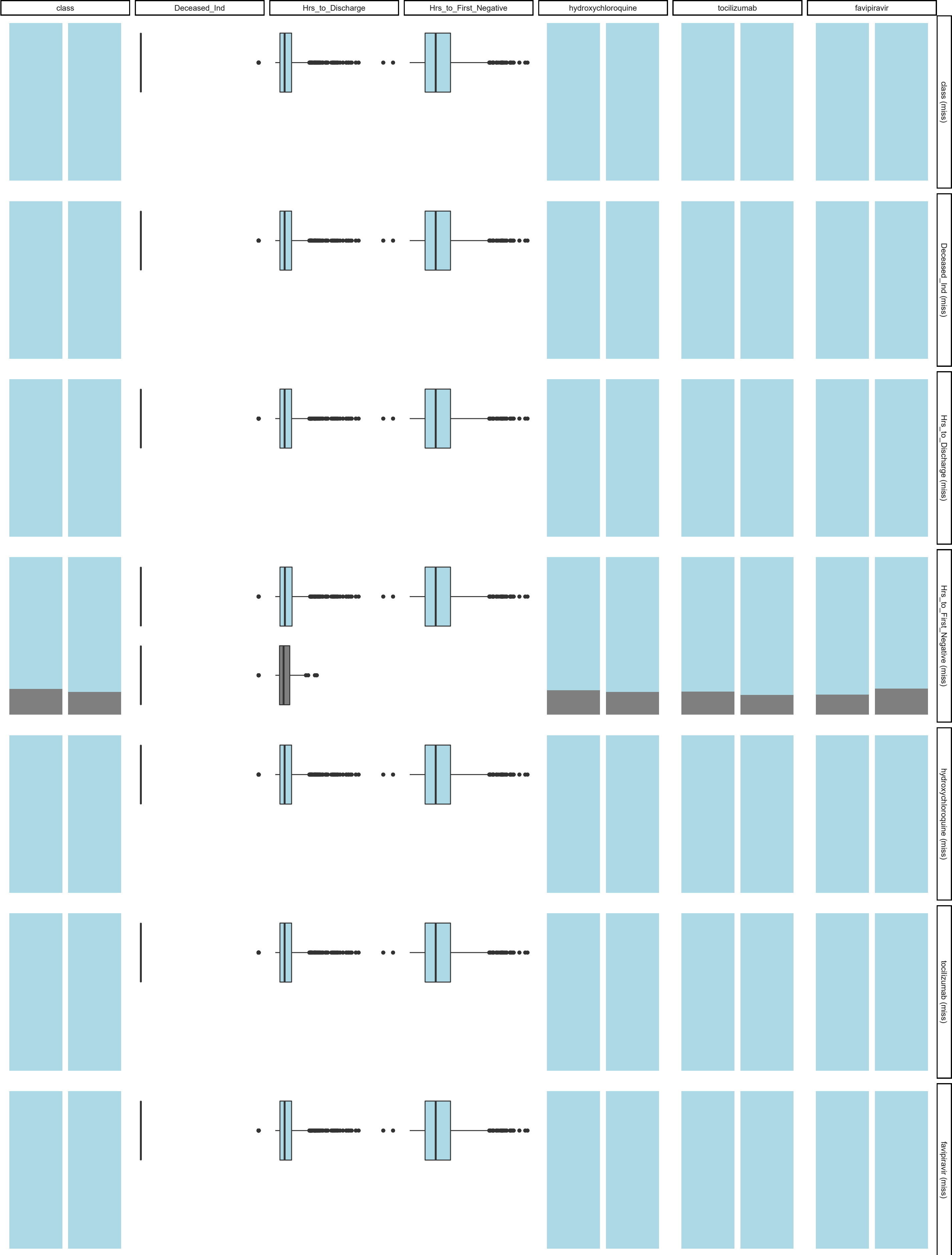

Supplement: S16 Fig — Explore patterns of missingness between levels of included variables. The pairs plots show relationships between missing values (gray) and observed values (Blue) for all the features. The distributions are used to visualize the continuous features, and the proportions are shown for categorical variables (continue). (PDF) [file pone.0291373.s016.pdf]

Missing data matrix

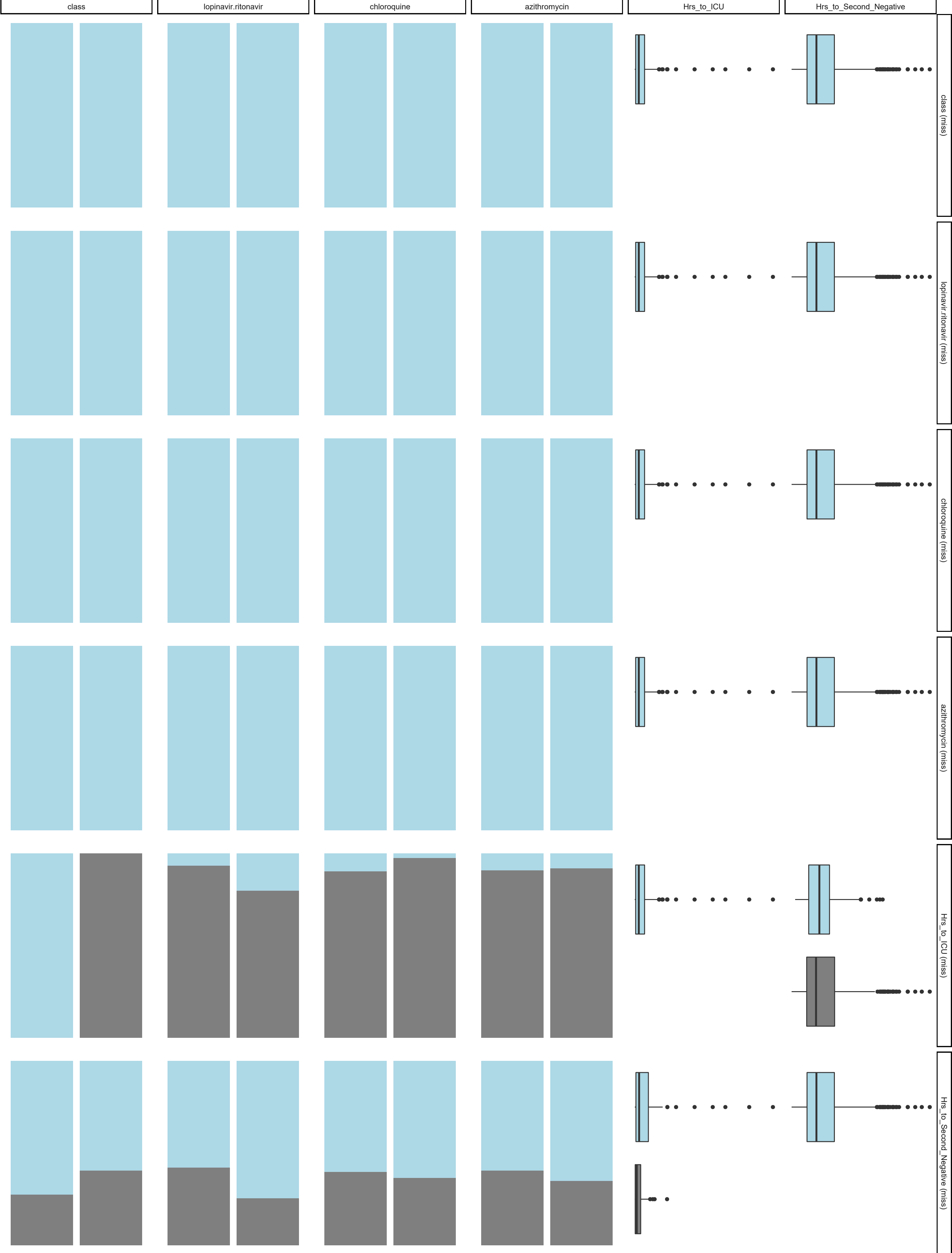

Supplement: S17 Fig — Explore patterns of missingness between levels of included variables. The pairs plots show relationships between missing values (gray) and observed values (Blue) for all the features. The distributions are used to visualize the continuous features, and the proportions are shown for categorical variables (continue). (PDF) [file pone.0291373.s017.pdf]

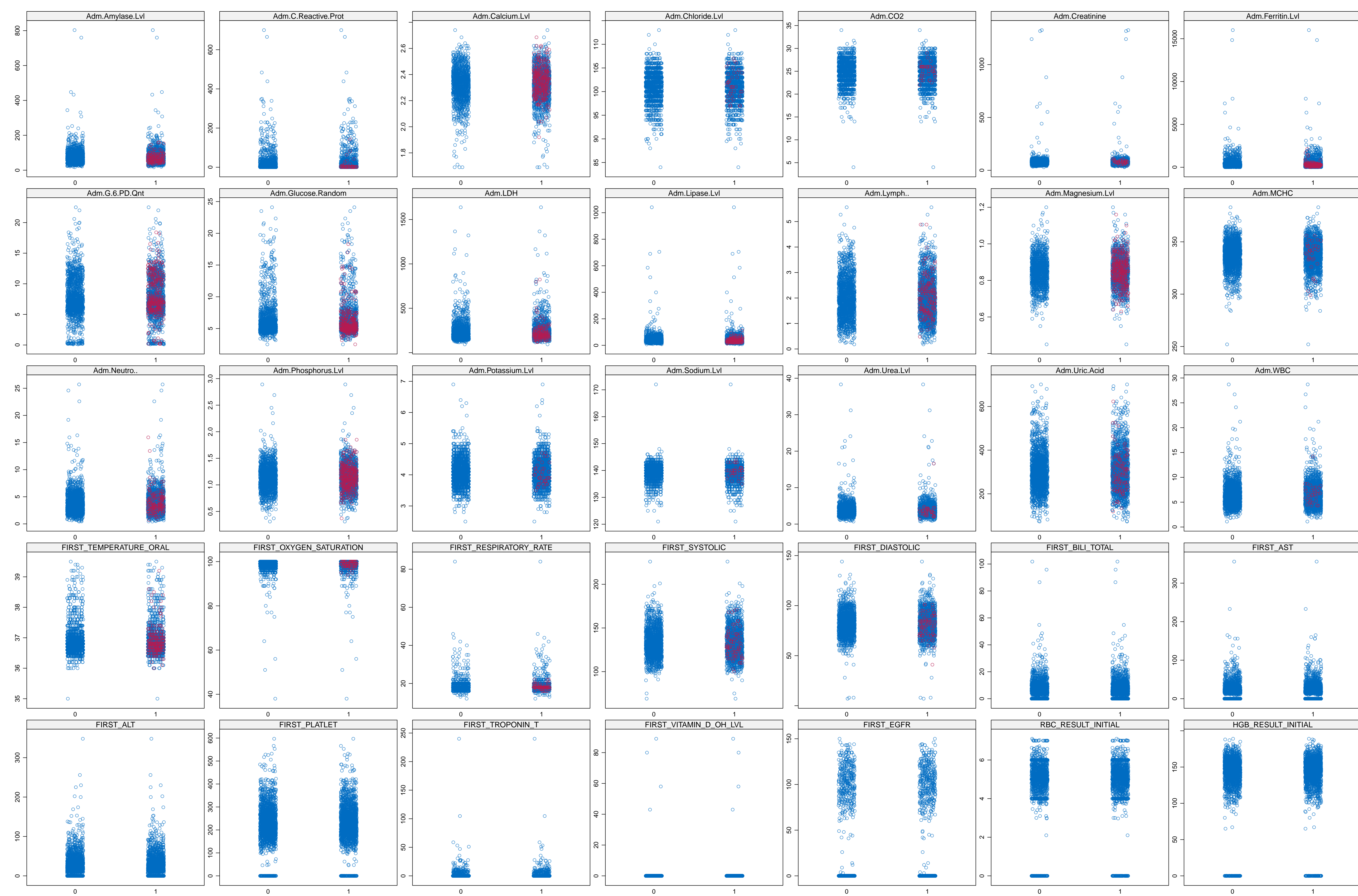

Supplement: S18 Fig — The figure compare the distribution of the original and imputed data. The magenta points represent the imputed points, and the blue ones show the observed ones. The plots infer that the imputed values are plausible values for the missing points. (PDF) [file pone.0291373.s018.pdf]

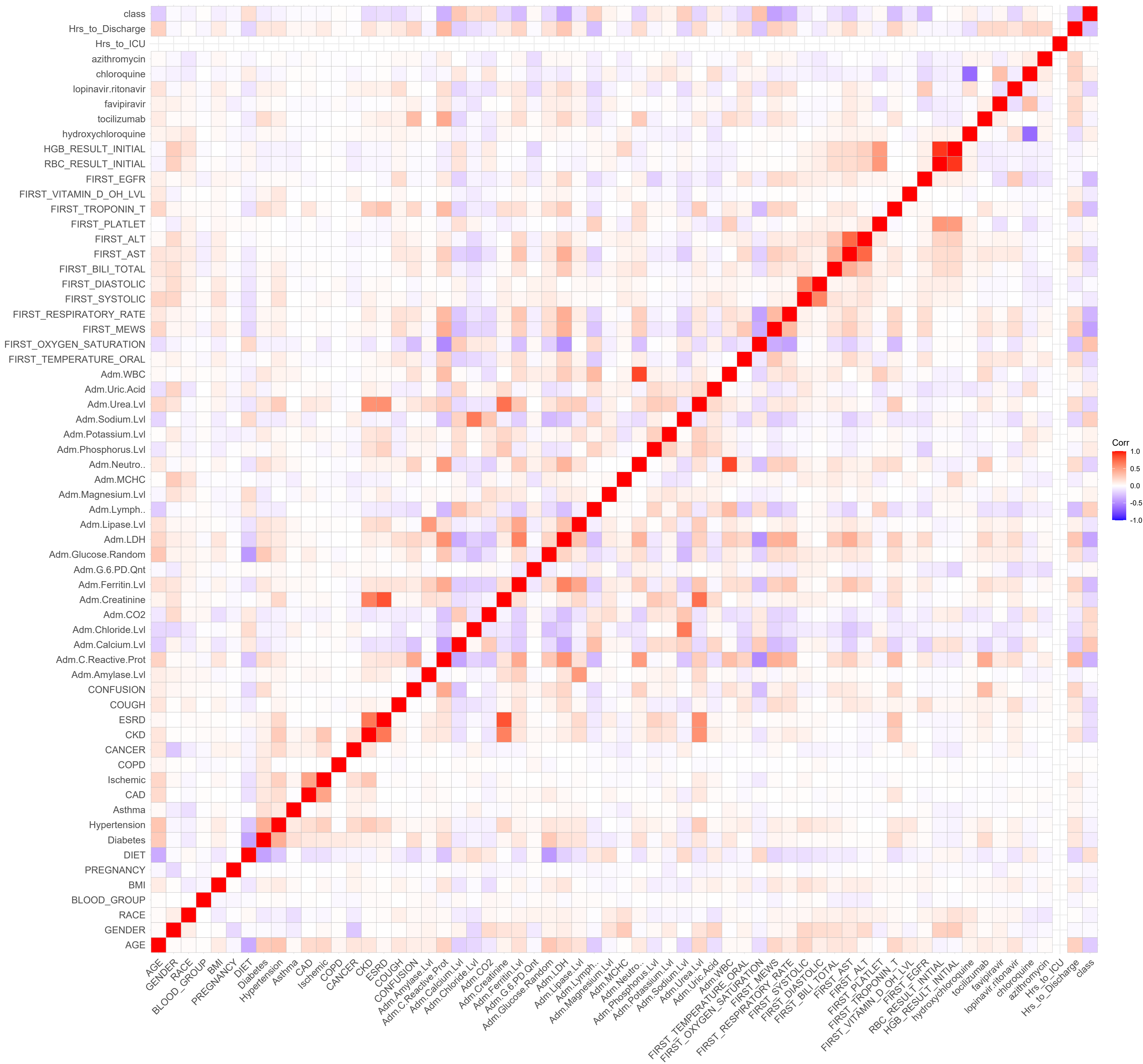

Supplement: S19 Fig — Heat map to visualize the correlation between the study features after removing the highly correlated features (〉0.7 and 〈-0.7). (PDF) [file pone.0291373.s019.pdf]
